# Supplementary material for: Epistatic mutations in PUMA BH3 drive an alternate binding mode to potently and selectively inhibit anti-apoptotic Bfl-1
Source: eLife. 2017 Jun 8;6:e25541. doi: 10.7554/eLife.25541 (PMC5464773; doi:10.7554/eLife.25541)
Supplement: Figure 3—source code 1. — DOI: http://dx.doi.org/10.7554/eLife.25541.014 [file elife-25541-fig3-code1.zip › elife-25541-fig3-code1-v1.html]

Figure 3 data analysis


In [12]:

```
%pylab inline
import pickle
import pandas as pd
import numpy as np
import matplotlib.pyplot as plt
from pandas import *
import seaborn as sns
```

```
Populating the interactive namespace from numpy and matplotlib
```

```
WARNING: pylab import has clobbered these variables: ['datetime', 'test', 'unique', 'info']
`%matplotlib` prevents importing * from pylab and numpy
```

In [13]:

```
# set graph styles
plt.style.use(['seaborn-white', 'seaborn-paper'])
matplotlib.rc("font", family="Arial")
sns.set_style("ticks")
sns.despine()
```

```
<matplotlib.figure.Figure at 0x11d956050>
```

In [14]:

```
# data sets of 100k ramdomly selected sequences from the indicated peptide library that were scored with STATIUM and SPOTpssm. STATIUM code and documentation is available at https://github.com/skoppula/statium 
F100klibZScores = pd.read_csv("/Users/jmjenson/Desktop/FSpaper/LibDesign/Bfl1100kZcomplete.txt")
X100klibZScores = pd.read_csv("/Users/jmjenson/Desktop/FSpaper/LibDesign/Bclxl100kZcomplete.txt")
M100klibZScores = pd.read_csv("/Users/jmjenson/Desktop/FSpaper/LibDesign/Mcl1100kZcomplete.txt")
# data set that includes PUMA scored with STATIUM and SPOTpssm
StandardsZScores = pd.read_csv("/Users/jmjenson/Desktop/FSpaper/LibDesign/testseq1Zcomplete.txt")
```

In [15]:

```
#load deep sequencing data sets 
comp1 = pd.read_csv("/Users/jmjenson/Documents/SORTCERY_003/comp1.csv",usecols=[1,2,3])
comp2 = pd.read_csv("/Users/jmjenson/Documents/SORTCERY_003/comp2.csv",usecols=[1,2,3])
comp3 = pd.read_csv("/Users/jmjenson/Documents/SORTCERY_003/comp3.csv",usecols=[1,2,3])
comp4 = pd.read_csv("/Users/jmjenson/Documents/SORTCERY_003/comp4.csv",usecols=[1,2,3])
pool = pd.read_csv("/Users/jmjenson/Documents/SORTCERY_003/pool.csv",usecols=[1,2,3])
compRedo3 = pd.read_csv("/Users/jmjenson/Documents/SORTCERY_003/compRedo3.csv",usecols=[1,2,3])
compRedo4 = pd.read_csv("/Users/jmjenson/Documents/SORTCERY_003/compRedo4.csv",usecols=[1,2,3])
compRedo4noStop=compRedo4[~compRedo4['seq'].str.contains("\*")] #filter out sequences with stop codons
#load STATIUM and SPOTpssm scores for sequences in the final pool (compRedo4)
CompRedo4NoStopScores = pd.read_csv("/Users/jmjenson/Desktop/FSpaper/LibDesign/compRedo4Zcomplete.txt")
CompRedo4NoStopScoresAdj= CompRedo4NoStopScores
#add counts from illumina sequencing to dataframe
CompRedo4NoStopScoresAdj['count']=compRedo4noStop['count']
```

In [16]:

```
#sum counts from illumina sequencing
sorts=[pool,comp1,comp2,comp3,comp4]
sortsRedo=[pool,comp1,comp2,compRedo3,compRedo4]

totreads_redo= []
for each in sortsRedo:
    totreads_redo.append(float(each['count'].sum()))
totreads_redo=np.array(totreads_redo)

Flib_1mut_redo=[]
for each in sortsRedo:
    Flib_1mut_redo.append(float(each[each['seq'].str.contains(".[A,E,I,K,L,P,Q,T,V]REI[A,C,D,G,S,Y]A[C,D,F,G,H,I,L,N,R,S,V,Y]LRR[A,C,F,G,I,L,P,R,S,T,V,]AD[D,E,H,I,K,L,M,N,Q,V][A,D,F,H,I,L,N,P,S,T,V,Y]NAQ[A,F,I,L,P,S,T,V]|W.REI[A,C,D,G,S,Y]A[C,D,F,G,H,I,L,N,R,S,V,Y]LRR[A,C,F,G,I,L,P,R,S,T,V,]AD[D,E,H,I,K,L,M,N,Q,V][A,D,F,H,I,L,N,P,S,T,V,Y]NAQ[A,F,I,L,P,S,T,V]|W[A,E,I,K,L,P,Q,T,V].EI[A,C,D,G,S,Y]A[C,D,F,G,H,I,L,N,R,S,V,Y]LRR[A,C,F,G,I,L,P,R,S,T,V,]AD[D,E,H,I,K,L,M,N,Q,V][A,D,F,H,I,L,N,P,S,T,V,Y]NAQ[A,F,I,L,P,S,T,V]|W[A,E,I,K,L,P,Q,T,V]R.I[A,C,D,G,S,Y]A[C,D,F,G,H,I,L,N,R,S,V,Y]LRR[A,C,F,G,I,L,P,R,S,T,V,]AD[D,E,H,I,K,L,M,N,Q,V][A,D,F,H,I,L,N,P,S,T,V,Y]NAQ[A,F,I,L,P,S,T,V]|W[A,E,I,K,L,P,Q,T,V]RE.[A,C,D,G,S,Y]A[C,D,F,G,H,I,L,N,R,S,V,Y]LRR[A,C,F,G,I,L,P,R,S,T,V,]AD[D,E,H,I,K,L,M,N,Q,V][A,D,F,H,I,L,N,P,S,T,V,Y]NAQ[A,F,I,L,P,S,T,V]|W[A,E,I,K,L,P,Q,T,V]REI.A[C,D,F,G,H,I,L,N,R,S,V,Y]LRR[A,C,F,G,I,L,P,R,S,T,V,]AD[D,E,H,I,K,L,M,N,Q,V][A,D,F,H,I,L,N,P,S,T,V,Y]NAQ[A,F,I,L,P,S,T,V]|W[A,E,I,K,L,P,Q,T,V]REI[A,C,D,G,S,Y].[C,D,F,G,H,I,L,N,R,S,V,Y]LRR[A,C,F,G,I,L,P,R,S,T,V,]AD[D,E,H,I,K,L,M,N,Q,V][A,D,F,H,I,L,N,P,S,T,V,Y]NAQ[A,F,I,L,P,S,T,V]|W[A,E,I,K,L,P,Q,T,V]REI[A,C,D,G,S,Y]A.LRR[A,C,F,G,I,L,P,R,S,T,V,]AD[D,E,H,I,K,L,M,N,Q,V][A,D,F,H,I,L,N,P,S,T,V,Y]NAQ[A,F,I,L,P,S,T,V]|W[A,E,I,K,L,P,Q,T,V]REI[A,C,D,G,S,Y]A[C,D,F,G,H,I,L,N,R,S,V,Y].RR[A,C,F,G,I,L,P,R,S,T,V,]AD[D,E,H,I,K,L,M,N,Q,V][A,D,F,H,I,L,N,P,S,T,V,Y]NAQ[A,F,I,L,P,S,T,V]|W[A,E,I,K,L,P,Q,T,V]REI[A,C,D,G,S,Y]A[C,D,F,G,H,I,L,N,R,S,V,Y]L.R[A,C,F,G,I,L,P,R,S,T,V,]AD[D,E,H,I,K,L,M,N,Q,V][A,D,F,H,I,L,N,P,S,T,V,Y]NAQ[A,F,I,L,P,S,T,V]|W[A,E,I,K,L,P,Q,T,V]REI[A,C,D,G,S,Y]A[C,D,F,G,H,I,L,N,R,S,V,Y]LR.[A,C,F,G,I,L,P,R,S,T,V,]AD[D,E,H,I,K,L,M,N,Q,V][A,D,F,H,I,L,N,P,S,T,V,Y]NAQ[A,F,I,L,P,S,T,V]|W[A,E,I,K,L,P,Q,T,V]REI[A,C,D,G,S,Y]A[C,D,F,G,H,I,L,N,R,S,V,Y]LRR.AD[D,E,H,I,K,L,M,N,Q,V][A,D,F,H,I,L,N,P,S,T,V,Y]NAQ[A,F,I,L,P,S,T,V]|W[A,E,I,K,L,P,Q,T,V]REI[A,C,D,G,S,Y]A[C,D,F,G,H,I,L,N,R,S,V,Y]LRR[A,C,F,G,I,L,P,R,S,T,V,].D[D,E,H,I,K,L,M,N,Q,V][A,D,F,H,I,L,N,P,S,T,V,Y]NAQ[A,F,I,L,P,S,T,V]|W[A,E,I,K,L,P,Q,T,V]REI[A,C,D,G,S,Y]A[C,D,F,G,H,I,L,N,R,S,V,Y]LRR[A,C,F,G,I,L,P,R,S,T,V,]A.[D,E,H,I,K,L,M,N,Q,V][A,D,F,H,I,L,N,P,S,T,V,Y]NAQ[A,F,I,L,P,S,T,V]|W[A,E,I,K,L,P,Q,T,V]REI[A,C,D,G,S,Y]A[C,D,F,G,H,I,L,N,R,S,V,Y]LRR[A,C,F,G,I,L,P,R,S,T,V,]AD.NAQ[A,F,I,L,P,S,T,V]|W[A,E,I,K,L,P,Q,T,V]REI[A,C,D,G,S,Y]A[C,D,F,G,H,I,L,N,R,S,V,Y]LRR[A,C,F,G,I,L,P,R,S,T,V,]AD[D,E,H,I,K,L,M,N,Q,V][A,D,F,H,I,L,N,P,S,T,V,Y].AQ[A,F,I,L,P,S,T,V]|W[A,E,I,K,L,P,Q,T,V]REI[A,C,D,G,S,Y]A[C,D,F,G,H,I,L,N,R,S,V,Y]LRR[A,C,F,G,I,L,P,R,S,T,V,]AD[D,E,H,I,K,L,M,N,Q,V][A,D,F,H,I,L,N,P,S,T,V,Y]N.Q[A,F,I,L,P,S,T,V]|W[A,E,I,K,L,P,Q,T,V]REI[A,C,D,G,S,Y]A[C,D,F,G,H,I,L,N,R,S,V,Y]LRR[A,C,F,G,I,L,P,R,S,T,V,]AD[D,E,H,I,K,L,M,N,Q,V][A,D,F,H,I,L,N,P,S,T,V,Y]NA.[A,F,I,L,P,S,T,V]|W[A,E,I,K,L,P,Q,T,V]REI[A,C,D,G,S,Y]A[C,D,F,G,H,I,L,N,R,S,V,Y]LRR[A,C,F,G,I,L,P,R,S,T,V,]AD[D,E,H,I,K,L,M,N,Q,V][A,D,F,H,I,L,N,P,S,T,V,Y]NAQ." 
                                                       )]['count'].sum()))
Flib_1mut_redo=np.array(Flib_1mut_redo)

Flib_1mut_Run1=[]
for each in sorts:
    Flib_1mut_Run1.append(float(each[each['seq'].str.contains(".[A,E,I,K,L,P,Q,T,V]REI[A,C,D,G,S,Y]A[C,D,F,G,H,I,L,N,R,S,V,Y]LRR[A,C,F,G,I,L,P,R,S,T,V,]AD[D,E,H,I,K,L,M,N,Q,V][A,D,F,H,I,L,N,P,S,T,V,Y]NAQ[A,F,I,L,P,S,T,V]|W.REI[A,C,D,G,S,Y]A[C,D,F,G,H,I,L,N,R,S,V,Y]LRR[A,C,F,G,I,L,P,R,S,T,V,]AD[D,E,H,I,K,L,M,N,Q,V][A,D,F,H,I,L,N,P,S,T,V,Y]NAQ[A,F,I,L,P,S,T,V]|W[A,E,I,K,L,P,Q,T,V].EI[A,C,D,G,S,Y]A[C,D,F,G,H,I,L,N,R,S,V,Y]LRR[A,C,F,G,I,L,P,R,S,T,V,]AD[D,E,H,I,K,L,M,N,Q,V][A,D,F,H,I,L,N,P,S,T,V,Y]NAQ[A,F,I,L,P,S,T,V]|W[A,E,I,K,L,P,Q,T,V]R.I[A,C,D,G,S,Y]A[C,D,F,G,H,I,L,N,R,S,V,Y]LRR[A,C,F,G,I,L,P,R,S,T,V,]AD[D,E,H,I,K,L,M,N,Q,V][A,D,F,H,I,L,N,P,S,T,V,Y]NAQ[A,F,I,L,P,S,T,V]|W[A,E,I,K,L,P,Q,T,V]RE.[A,C,D,G,S,Y]A[C,D,F,G,H,I,L,N,R,S,V,Y]LRR[A,C,F,G,I,L,P,R,S,T,V,]AD[D,E,H,I,K,L,M,N,Q,V][A,D,F,H,I,L,N,P,S,T,V,Y]NAQ[A,F,I,L,P,S,T,V]|W[A,E,I,K,L,P,Q,T,V]REI.A[C,D,F,G,H,I,L,N,R,S,V,Y]LRR[A,C,F,G,I,L,P,R,S,T,V,]AD[D,E,H,I,K,L,M,N,Q,V][A,D,F,H,I,L,N,P,S,T,V,Y]NAQ[A,F,I,L,P,S,T,V]|W[A,E,I,K,L,P,Q,T,V]REI[A,C,D,G,S,Y].[C,D,F,G,H,I,L,N,R,S,V,Y]LRR[A,C,F,G,I,L,P,R,S,T,V,]AD[D,E,H,I,K,L,M,N,Q,V][A,D,F,H,I,L,N,P,S,T,V,Y]NAQ[A,F,I,L,P,S,T,V]|W[A,E,I,K,L,P,Q,T,V]REI[A,C,D,G,S,Y]A.LRR[A,C,F,G,I,L,P,R,S,T,V,]AD[D,E,H,I,K,L,M,N,Q,V][A,D,F,H,I,L,N,P,S,T,V,Y]NAQ[A,F,I,L,P,S,T,V]|W[A,E,I,K,L,P,Q,T,V]REI[A,C,D,G,S,Y]A[C,D,F,G,H,I,L,N,R,S,V,Y].RR[A,C,F,G,I,L,P,R,S,T,V,]AD[D,E,H,I,K,L,M,N,Q,V][A,D,F,H,I,L,N,P,S,T,V,Y]NAQ[A,F,I,L,P,S,T,V]|W[A,E,I,K,L,P,Q,T,V]REI[A,C,D,G,S,Y]A[C,D,F,G,H,I,L,N,R,S,V,Y]L.R[A,C,F,G,I,L,P,R,S,T,V,]AD[D,E,H,I,K,L,M,N,Q,V][A,D,F,H,I,L,N,P,S,T,V,Y]NAQ[A,F,I,L,P,S,T,V]|W[A,E,I,K,L,P,Q,T,V]REI[A,C,D,G,S,Y]A[C,D,F,G,H,I,L,N,R,S,V,Y]LR.[A,C,F,G,I,L,P,R,S,T,V,]AD[D,E,H,I,K,L,M,N,Q,V][A,D,F,H,I,L,N,P,S,T,V,Y]NAQ[A,F,I,L,P,S,T,V]|W[A,E,I,K,L,P,Q,T,V]REI[A,C,D,G,S,Y]A[C,D,F,G,H,I,L,N,R,S,V,Y]LRR.AD[D,E,H,I,K,L,M,N,Q,V][A,D,F,H,I,L,N,P,S,T,V,Y]NAQ[A,F,I,L,P,S,T,V]|W[A,E,I,K,L,P,Q,T,V]REI[A,C,D,G,S,Y]A[C,D,F,G,H,I,L,N,R,S,V,Y]LRR[A,C,F,G,I,L,P,R,S,T,V,].D[D,E,H,I,K,L,M,N,Q,V][A,D,F,H,I,L,N,P,S,T,V,Y]NAQ[A,F,I,L,P,S,T,V]|W[A,E,I,K,L,P,Q,T,V]REI[A,C,D,G,S,Y]A[C,D,F,G,H,I,L,N,R,S,V,Y]LRR[A,C,F,G,I,L,P,R,S,T,V,]A.[D,E,H,I,K,L,M,N,Q,V][A,D,F,H,I,L,N,P,S,T,V,Y]NAQ[A,F,I,L,P,S,T,V]|W[A,E,I,K,L,P,Q,T,V]REI[A,C,D,G,S,Y]A[C,D,F,G,H,I,L,N,R,S,V,Y]LRR[A,C,F,G,I,L,P,R,S,T,V,]AD.NAQ[A,F,I,L,P,S,T,V]|W[A,E,I,K,L,P,Q,T,V]REI[A,C,D,G,S,Y]A[C,D,F,G,H,I,L,N,R,S,V,Y]LRR[A,C,F,G,I,L,P,R,S,T,V,]AD[D,E,H,I,K,L,M,N,Q,V][A,D,F,H,I,L,N,P,S,T,V,Y].AQ[A,F,I,L,P,S,T,V]|W[A,E,I,K,L,P,Q,T,V]REI[A,C,D,G,S,Y]A[C,D,F,G,H,I,L,N,R,S,V,Y]LRR[A,C,F,G,I,L,P,R,S,T,V,]AD[D,E,H,I,K,L,M,N,Q,V][A,D,F,H,I,L,N,P,S,T,V,Y]N.Q[A,F,I,L,P,S,T,V]|W[A,E,I,K,L,P,Q,T,V]REI[A,C,D,G,S,Y]A[C,D,F,G,H,I,L,N,R,S,V,Y]LRR[A,C,F,G,I,L,P,R,S,T,V,]AD[D,E,H,I,K,L,M,N,Q,V][A,D,F,H,I,L,N,P,S,T,V,Y]NA.[A,F,I,L,P,S,T,V]|W[A,E,I,K,L,P,Q,T,V]REI[A,C,D,G,S,Y]A[C,D,F,G,H,I,L,N,R,S,V,Y]LRR[A,C,F,G,I,L,P,R,S,T,V,]AD[D,E,H,I,K,L,M,N,Q,V][A,D,F,H,I,L,N,P,S,T,V,Y]NAQ." 
                                                       )]['count'].sum()))
Flib_1mut_Run1=np.array(Flib_1mut_Run1)

Flib_1mut_Run1C=[]
for each in sorts:
    Flib_1mut_Run1C.append(float(each[each['seq'].str.contains("C")&each['seq'].str.contains(".[A,E,I,K,L,P,Q,T,V]REI[A,C,D,G,S,Y]A[C,D,F,G,H,I,L,N,R,S,V,Y]LRR[A,C,F,G,I,L,P,R,S,T,V,]AD[D,E,H,I,K,L,M,N,Q,V][A,D,F,H,I,L,N,P,S,T,V,Y]NAQ[A,F,I,L,P,S,T,V]|W.REI[A,C,D,G,S,Y]A[C,D,F,G,H,I,L,N,R,S,V,Y]LRR[A,C,F,G,I,L,P,R,S,T,V,]AD[D,E,H,I,K,L,M,N,Q,V][A,D,F,H,I,L,N,P,S,T,V,Y]NAQ[A,F,I,L,P,S,T,V]|W[A,E,I,K,L,P,Q,T,V].EI[A,C,D,G,S,Y]A[C,D,F,G,H,I,L,N,R,S,V,Y]LRR[A,C,F,G,I,L,P,R,S,T,V,]AD[D,E,H,I,K,L,M,N,Q,V][A,D,F,H,I,L,N,P,S,T,V,Y]NAQ[A,F,I,L,P,S,T,V]|W[A,E,I,K,L,P,Q,T,V]R.I[A,C,D,G,S,Y]A[C,D,F,G,H,I,L,N,R,S,V,Y]LRR[A,C,F,G,I,L,P,R,S,T,V,]AD[D,E,H,I,K,L,M,N,Q,V][A,D,F,H,I,L,N,P,S,T,V,Y]NAQ[A,F,I,L,P,S,T,V]|W[A,E,I,K,L,P,Q,T,V]RE.[A,C,D,G,S,Y]A[C,D,F,G,H,I,L,N,R,S,V,Y]LRR[A,C,F,G,I,L,P,R,S,T,V,]AD[D,E,H,I,K,L,M,N,Q,V][A,D,F,H,I,L,N,P,S,T,V,Y]NAQ[A,F,I,L,P,S,T,V]|W[A,E,I,K,L,P,Q,T,V]REI.A[C,D,F,G,H,I,L,N,R,S,V,Y]LRR[A,C,F,G,I,L,P,R,S,T,V,]AD[D,E,H,I,K,L,M,N,Q,V][A,D,F,H,I,L,N,P,S,T,V,Y]NAQ[A,F,I,L,P,S,T,V]|W[A,E,I,K,L,P,Q,T,V]REI[A,C,D,G,S,Y].[C,D,F,G,H,I,L,N,R,S,V,Y]LRR[A,C,F,G,I,L,P,R,S,T,V,]AD[D,E,H,I,K,L,M,N,Q,V][A,D,F,H,I,L,N,P,S,T,V,Y]NAQ[A,F,I,L,P,S,T,V]|W[A,E,I,K,L,P,Q,T,V]REI[A,C,D,G,S,Y]A.LRR[A,C,F,G,I,L,P,R,S,T,V,]AD[D,E,H,I,K,L,M,N,Q,V][A,D,F,H,I,L,N,P,S,T,V,Y]NAQ[A,F,I,L,P,S,T,V]|W[A,E,I,K,L,P,Q,T,V]REI[A,C,D,G,S,Y]A[C,D,F,G,H,I,L,N,R,S,V,Y].RR[A,C,F,G,I,L,P,R,S,T,V,]AD[D,E,H,I,K,L,M,N,Q,V][A,D,F,H,I,L,N,P,S,T,V,Y]NAQ[A,F,I,L,P,S,T,V]|W[A,E,I,K,L,P,Q,T,V]REI[A,C,D,G,S,Y]A[C,D,F,G,H,I,L,N,R,S,V,Y]L.R[A,C,F,G,I,L,P,R,S,T,V,]AD[D,E,H,I,K,L,M,N,Q,V][A,D,F,H,I,L,N,P,S,T,V,Y]NAQ[A,F,I,L,P,S,T,V]|W[A,E,I,K,L,P,Q,T,V]REI[A,C,D,G,S,Y]A[C,D,F,G,H,I,L,N,R,S,V,Y]LR.[A,C,F,G,I,L,P,R,S,T,V,]AD[D,E,H,I,K,L,M,N,Q,V][A,D,F,H,I,L,N,P,S,T,V,Y]NAQ[A,F,I,L,P,S,T,V]|W[A,E,I,K,L,P,Q,T,V]REI[A,C,D,G,S,Y]A[C,D,F,G,H,I,L,N,R,S,V,Y]LRR.AD[D,E,H,I,K,L,M,N,Q,V][A,D,F,H,I,L,N,P,S,T,V,Y]NAQ[A,F,I,L,P,S,T,V]|W[A,E,I,K,L,P,Q,T,V]REI[A,C,D,G,S,Y]A[C,D,F,G,H,I,L,N,R,S,V,Y]LRR[A,C,F,G,I,L,P,R,S,T,V,].D[D,E,H,I,K,L,M,N,Q,V][A,D,F,H,I,L,N,P,S,T,V,Y]NAQ[A,F,I,L,P,S,T,V]|W[A,E,I,K,L,P,Q,T,V]REI[A,C,D,G,S,Y]A[C,D,F,G,H,I,L,N,R,S,V,Y]LRR[A,C,F,G,I,L,P,R,S,T,V,]A.[D,E,H,I,K,L,M,N,Q,V][A,D,F,H,I,L,N,P,S,T,V,Y]NAQ[A,F,I,L,P,S,T,V]|W[A,E,I,K,L,P,Q,T,V]REI[A,C,D,G,S,Y]A[C,D,F,G,H,I,L,N,R,S,V,Y]LRR[A,C,F,G,I,L,P,R,S,T,V,]AD.NAQ[A,F,I,L,P,S,T,V]|W[A,E,I,K,L,P,Q,T,V]REI[A,C,D,G,S,Y]A[C,D,F,G,H,I,L,N,R,S,V,Y]LRR[A,C,F,G,I,L,P,R,S,T,V,]AD[D,E,H,I,K,L,M,N,Q,V][A,D,F,H,I,L,N,P,S,T,V,Y].AQ[A,F,I,L,P,S,T,V]|W[A,E,I,K,L,P,Q,T,V]REI[A,C,D,G,S,Y]A[C,D,F,G,H,I,L,N,R,S,V,Y]LRR[A,C,F,G,I,L,P,R,S,T,V,]AD[D,E,H,I,K,L,M,N,Q,V][A,D,F,H,I,L,N,P,S,T,V,Y]N.Q[A,F,I,L,P,S,T,V]|W[A,E,I,K,L,P,Q,T,V]REI[A,C,D,G,S,Y]A[C,D,F,G,H,I,L,N,R,S,V,Y]LRR[A,C,F,G,I,L,P,R,S,T,V,]AD[D,E,H,I,K,L,M,N,Q,V][A,D,F,H,I,L,N,P,S,T,V,Y]NA.[A,F,I,L,P,S,T,V]|W[A,E,I,K,L,P,Q,T,V]REI[A,C,D,G,S,Y]A[C,D,F,G,H,I,L,N,R,S,V,Y]LRR[A,C,F,G,I,L,P,R,S,T,V,]AD[D,E,H,I,K,L,M,N,Q,V][A,D,F,H,I,L,N,P,S,T,V,Y]NAQ." 
                                                       )]['count'].sum()))
Flib_1mut_Run1C=np.array(Flib_1mut_Run1C)

Flib_1mut_RedoC=[]
for each in sortsRedo:
    Flib_1mut_RedoC.append(float(each[each['seq'].str.contains("C")&each['seq'].str.contains(".[A,E,I,K,L,P,Q,T,V]REI[A,C,D,G,S,Y]A[C,D,F,G,H,I,L,N,R,S,V,Y]LRR[A,C,F,G,I,L,P,R,S,T,V,]AD[D,E,H,I,K,L,M,N,Q,V][A,D,F,H,I,L,N,P,S,T,V,Y]NAQ[A,F,I,L,P,S,T,V]|W.REI[A,C,D,G,S,Y]A[C,D,F,G,H,I,L,N,R,S,V,Y]LRR[A,C,F,G,I,L,P,R,S,T,V,]AD[D,E,H,I,K,L,M,N,Q,V][A,D,F,H,I,L,N,P,S,T,V,Y]NAQ[A,F,I,L,P,S,T,V]|W[A,E,I,K,L,P,Q,T,V].EI[A,C,D,G,S,Y]A[C,D,F,G,H,I,L,N,R,S,V,Y]LRR[A,C,F,G,I,L,P,R,S,T,V,]AD[D,E,H,I,K,L,M,N,Q,V][A,D,F,H,I,L,N,P,S,T,V,Y]NAQ[A,F,I,L,P,S,T,V]|W[A,E,I,K,L,P,Q,T,V]R.I[A,C,D,G,S,Y]A[C,D,F,G,H,I,L,N,R,S,V,Y]LRR[A,C,F,G,I,L,P,R,S,T,V,]AD[D,E,H,I,K,L,M,N,Q,V][A,D,F,H,I,L,N,P,S,T,V,Y]NAQ[A,F,I,L,P,S,T,V]|W[A,E,I,K,L,P,Q,T,V]RE.[A,C,D,G,S,Y]A[C,D,F,G,H,I,L,N,R,S,V,Y]LRR[A,C,F,G,I,L,P,R,S,T,V,]AD[D,E,H,I,K,L,M,N,Q,V][A,D,F,H,I,L,N,P,S,T,V,Y]NAQ[A,F,I,L,P,S,T,V]|W[A,E,I,K,L,P,Q,T,V]REI.A[C,D,F,G,H,I,L,N,R,S,V,Y]LRR[A,C,F,G,I,L,P,R,S,T,V,]AD[D,E,H,I,K,L,M,N,Q,V][A,D,F,H,I,L,N,P,S,T,V,Y]NAQ[A,F,I,L,P,S,T,V]|W[A,E,I,K,L,P,Q,T,V]REI[A,C,D,G,S,Y].[C,D,F,G,H,I,L,N,R,S,V,Y]LRR[A,C,F,G,I,L,P,R,S,T,V,]AD[D,E,H,I,K,L,M,N,Q,V][A,D,F,H,I,L,N,P,S,T,V,Y]NAQ[A,F,I,L,P,S,T,V]|W[A,E,I,K,L,P,Q,T,V]REI[A,C,D,G,S,Y]A.LRR[A,C,F,G,I,L,P,R,S,T,V,]AD[D,E,H,I,K,L,M,N,Q,V][A,D,F,H,I,L,N,P,S,T,V,Y]NAQ[A,F,I,L,P,S,T,V]|W[A,E,I,K,L,P,Q,T,V]REI[A,C,D,G,S,Y]A[C,D,F,G,H,I,L,N,R,S,V,Y].RR[A,C,F,G,I,L,P,R,S,T,V,]AD[D,E,H,I,K,L,M,N,Q,V][A,D,F,H,I,L,N,P,S,T,V,Y]NAQ[A,F,I,L,P,S,T,V]|W[A,E,I,K,L,P,Q,T,V]REI[A,C,D,G,S,Y]A[C,D,F,G,H,I,L,N,R,S,V,Y]L.R[A,C,F,G,I,L,P,R,S,T,V,]AD[D,E,H,I,K,L,M,N,Q,V][A,D,F,H,I,L,N,P,S,T,V,Y]NAQ[A,F,I,L,P,S,T,V]|W[A,E,I,K,L,P,Q,T,V]REI[A,C,D,G,S,Y]A[C,D,F,G,H,I,L,N,R,S,V,Y]LR.[A,C,F,G,I,L,P,R,S,T,V,]AD[D,E,H,I,K,L,M,N,Q,V][A,D,F,H,I,L,N,P,S,T,V,Y]NAQ[A,F,I,L,P,S,T,V]|W[A,E,I,K,L,P,Q,T,V]REI[A,C,D,G,S,Y]A[C,D,F,G,H,I,L,N,R,S,V,Y]LRR.AD[D,E,H,I,K,L,M,N,Q,V][A,D,F,H,I,L,N,P,S,T,V,Y]NAQ[A,F,I,L,P,S,T,V]|W[A,E,I,K,L,P,Q,T,V]REI[A,C,D,G,S,Y]A[C,D,F,G,H,I,L,N,R,S,V,Y]LRR[A,C,F,G,I,L,P,R,S,T,V,].D[D,E,H,I,K,L,M,N,Q,V][A,D,F,H,I,L,N,P,S,T,V,Y]NAQ[A,F,I,L,P,S,T,V]|W[A,E,I,K,L,P,Q,T,V]REI[A,C,D,G,S,Y]A[C,D,F,G,H,I,L,N,R,S,V,Y]LRR[A,C,F,G,I,L,P,R,S,T,V,]A.[D,E,H,I,K,L,M,N,Q,V][A,D,F,H,I,L,N,P,S,T,V,Y]NAQ[A,F,I,L,P,S,T,V]|W[A,E,I,K,L,P,Q,T,V]REI[A,C,D,G,S,Y]A[C,D,F,G,H,I,L,N,R,S,V,Y]LRR[A,C,F,G,I,L,P,R,S,T,V,]AD.NAQ[A,F,I,L,P,S,T,V]|W[A,E,I,K,L,P,Q,T,V]REI[A,C,D,G,S,Y]A[C,D,F,G,H,I,L,N,R,S,V,Y]LRR[A,C,F,G,I,L,P,R,S,T,V,]AD[D,E,H,I,K,L,M,N,Q,V][A,D,F,H,I,L,N,P,S,T,V,Y].AQ[A,F,I,L,P,S,T,V]|W[A,E,I,K,L,P,Q,T,V]REI[A,C,D,G,S,Y]A[C,D,F,G,H,I,L,N,R,S,V,Y]LRR[A,C,F,G,I,L,P,R,S,T,V,]AD[D,E,H,I,K,L,M,N,Q,V][A,D,F,H,I,L,N,P,S,T,V,Y]N.Q[A,F,I,L,P,S,T,V]|W[A,E,I,K,L,P,Q,T,V]REI[A,C,D,G,S,Y]A[C,D,F,G,H,I,L,N,R,S,V,Y]LRR[A,C,F,G,I,L,P,R,S,T,V,]AD[D,E,H,I,K,L,M,N,Q,V][A,D,F,H,I,L,N,P,S,T,V,Y]NA.[A,F,I,L,P,S,T,V]|W[A,E,I,K,L,P,Q,T,V]REI[A,C,D,G,S,Y]A[C,D,F,G,H,I,L,N,R,S,V,Y]LRR[A,C,F,G,I,L,P,R,S,T,V,]AD[D,E,H,I,K,L,M,N,Q,V][A,D,F,H,I,L,N,P,S,T,V,Y]NAQ." 
                                                       )]['count'].sum()))
Flib_1mut_RedoC=np.array(Flib_1mut_RedoC)


Xlib_1mut_redo=[]
for each in sortsRedo:
    Xlib_1mut_redo.append(float(each[each['seq'].str.contains(".[DEHIKLMNQV]RE[DFHILNVY]GAQL[AGIRTV]R[ACDFGHILNPRSTVY][AG]DDL[ADHILNPTV][AEKT]Q[ACDGHNPRSTY]|W.RE[DFHILNVY]GAQL[AGIRTV]R[ACDFGHILNPRSTVY][AG]DDL[ADHILNPTV][AEKT]Q[ACDGHNPRSTY]|W[DEHIKLMNQV].E[DFHILNVY]GAQL[AGIRTV]R[ACDFGHILNPRSTVY][AG]DDL[ADHILNPTV][AEKT]Q[ACDGHNPRSTY]|W[DEHIKLMNQV]R.[DFHILNVY]GAQL[AGIRTV]R[ACDFGHILNPRSTVY][AG]DDL[ADHILNPTV][AEKT]Q[ACDGHNPRSTY]|W[DEHIKLMNQV]RE.GAQL[AGIRTV]R[ACDFGHILNPRSTVY][AG]DDL[ADHILNPTV][AEKT]Q[ACDGHNPRSTY]|W[DEHIKLMNQV]RE[DFHILNVY].AQL[AGIRTV]R[ACDFGHILNPRSTVY][AG]DDL[ADHILNPTV][AEKT]Q[ACDGHNPRSTY]|W[DEHIKLMNQV]RE[DFHILNVY]G.QL[AGIRTV]R[ACDFGHILNPRSTVY][AG]DDL[ADHILNPTV][AEKT]Q[ACDGHNPRSTY]|W[DEHIKLMNQV]RE[DFHILNVY]GA.L[AGIRTV]R[ACDFGHILNPRSTVY][AG]DDL[ADHILNPTV][AEKT]Q[ACDGHNPRSTY]|W[DEHIKLMNQV]RE[DFHILNVY]GAQ.[AGIRTV]R[ACDFGHILNPRSTVY][AG]DDL[ADHILNPTV][AEKT]Q[ACDGHNPRSTY]|W[DEHIKLMNQV]RE[DFHILNVY]GAQL.R[ACDFGHILNPRSTVY][AG]DDL[ADHILNPTV][AEKT]Q[ACDGHNPRSTY]|W[DEHIKLMNQV]RE[DFHILNVY]GAQL[AGIRTV].[ACDFGHILNPRSTVY][AG]DDL[ADHILNPTV][AEKT]Q[ACDGHNPRSTY]|W[DEHIKLMNQV]RE[DFHILNVY]GAQL[AGIRTV]R.[AG]DDL[ADHILNPTV][AEKT]Q[ACDGHNPRSTY]|W[DEHIKLMNQV]RE[DFHILNVY]GAQL[AGIRTV]R[ACDFGHILNPRSTVY].DDL[ADHILNPTV][AEKT]Q[ACDGHNPRSTY]|W[DEHIKLMNQV]RE[DFHILNVY]GAQL[AGIRTV]R[ACDFGHILNPRSTVY][AG].DL[ADHILNPTV][AEKT]Q[ACDGHNPRSTY]|W[DEHIKLMNQV]RE[DFHILNVY]GAQL[AGIRTV]R[ACDFGHILNPRSTVY][AG]D.L[ADHILNPTV][AEKT]Q[ACDGHNPRSTY]|W[DEHIKLMNQV]RE[DFHILNVY]GAQL[AGIRTV]R[ACDFGHILNPRSTVY][AG]DD.[ADHILNPTV][AEKT]Q[ACDGHNPRSTY]|W[DEHIKLMNQV]RE[DFHILNVY]GAQL[AGIRTV]R[ACDFGHILNPRSTVY][AG]DDL.[AEKT]Q[ACDGHNPRSTY]|W[DEHIKLMNQV]RE[DFHILNVY]GAQL[AGIRTV]R[ACDFGHILNPRSTVY][AG]DDL[ADHILNPTV].Q[ACDGHNPRSTY]|W[DEHIKLMNQV]RE[DFHILNVY]GAQL[AGIRTV]R[ACDFGHILNPRSTVY][AG]DDL[ADHILNPTV][AEKT].[ACDGHNPRSTY]|W[DEHIKLMNQV]RE[DFHILNVY]GAQL[AGIRTV]R[ACDFGHILNPRSTVY][AG]DDL[ADHILNPTV][AEKT]Q."    
                                                        )]['count'].sum()))
Xlib_1mut_redo=np.array(Xlib_1mut_redo)

Xlib_1mut_Run1=[]
for each in sorts:
    Xlib_1mut_Run1.append(float(each[each['seq'].str.contains(".[DEHIKLMNQV]RE[DFHILNVY]GAQL[AGIRTV]R[ACDFGHILNPRSTVY][AG]DDL[ADHILNPTV][AEKT]Q[ACDGHNPRSTY]|W.RE[DFHILNVY]GAQL[AGIRTV]R[ACDFGHILNPRSTVY][AG]DDL[ADHILNPTV][AEKT]Q[ACDGHNPRSTY]|W[DEHIKLMNQV].E[DFHILNVY]GAQL[AGIRTV]R[ACDFGHILNPRSTVY][AG]DDL[ADHILNPTV][AEKT]Q[ACDGHNPRSTY]|W[DEHIKLMNQV]R.[DFHILNVY]GAQL[AGIRTV]R[ACDFGHILNPRSTVY][AG]DDL[ADHILNPTV][AEKT]Q[ACDGHNPRSTY]|W[DEHIKLMNQV]RE.GAQL[AGIRTV]R[ACDFGHILNPRSTVY][AG]DDL[ADHILNPTV][AEKT]Q[ACDGHNPRSTY]|W[DEHIKLMNQV]RE[DFHILNVY].AQL[AGIRTV]R[ACDFGHILNPRSTVY][AG]DDL[ADHILNPTV][AEKT]Q[ACDGHNPRSTY]|W[DEHIKLMNQV]RE[DFHILNVY]G.QL[AGIRTV]R[ACDFGHILNPRSTVY][AG]DDL[ADHILNPTV][AEKT]Q[ACDGHNPRSTY]|W[DEHIKLMNQV]RE[DFHILNVY]GA.L[AGIRTV]R[ACDFGHILNPRSTVY][AG]DDL[ADHILNPTV][AEKT]Q[ACDGHNPRSTY]|W[DEHIKLMNQV]RE[DFHILNVY]GAQ.[AGIRTV]R[ACDFGHILNPRSTVY][AG]DDL[ADHILNPTV][AEKT]Q[ACDGHNPRSTY]|W[DEHIKLMNQV]RE[DFHILNVY]GAQL.R[ACDFGHILNPRSTVY][AG]DDL[ADHILNPTV][AEKT]Q[ACDGHNPRSTY]|W[DEHIKLMNQV]RE[DFHILNVY]GAQL[AGIRTV].[ACDFGHILNPRSTVY][AG]DDL[ADHILNPTV][AEKT]Q[ACDGHNPRSTY]|W[DEHIKLMNQV]RE[DFHILNVY]GAQL[AGIRTV]R.[AG]DDL[ADHILNPTV][AEKT]Q[ACDGHNPRSTY]|W[DEHIKLMNQV]RE[DFHILNVY]GAQL[AGIRTV]R[ACDFGHILNPRSTVY].DDL[ADHILNPTV][AEKT]Q[ACDGHNPRSTY]|W[DEHIKLMNQV]RE[DFHILNVY]GAQL[AGIRTV]R[ACDFGHILNPRSTVY][AG].DL[ADHILNPTV][AEKT]Q[ACDGHNPRSTY]|W[DEHIKLMNQV]RE[DFHILNVY]GAQL[AGIRTV]R[ACDFGHILNPRSTVY][AG]D.L[ADHILNPTV][AEKT]Q[ACDGHNPRSTY]|W[DEHIKLMNQV]RE[DFHILNVY]GAQL[AGIRTV]R[ACDFGHILNPRSTVY][AG]DD.[ADHILNPTV][AEKT]Q[ACDGHNPRSTY]|W[DEHIKLMNQV]RE[DFHILNVY]GAQL[AGIRTV]R[ACDFGHILNPRSTVY][AG]DDL.[AEKT]Q[ACDGHNPRSTY]|W[DEHIKLMNQV]RE[DFHILNVY]GAQL[AGIRTV]R[ACDFGHILNPRSTVY][AG]DDL[ADHILNPTV].Q[ACDGHNPRSTY]|W[DEHIKLMNQV]RE[DFHILNVY]GAQL[AGIRTV]R[ACDFGHILNPRSTVY][AG]DDL[ADHILNPTV][AEKT].[ACDGHNPRSTY]|W[DEHIKLMNQV]RE[DFHILNVY]GAQL[AGIRTV]R[ACDFGHILNPRSTVY][AG]DDL[ADHILNPTV][AEKT]Q."    
                                                        )]['count'].sum()))
Xlib_1mut_Run1=np.array(Xlib_1mut_Run1)

Xlib_1mut_Run1C=[]
for each in sorts:
    Xlib_1mut_Run1C.append(float(each[each['seq'].str.contains("C")&each['seq'].str.contains(".[DEHIKLMNQV]RE[DFHILNVY]GAQL[AGIRTV]R[ACDFGHILNPRSTVY][AG]DDL[ADHILNPTV][AEKT]Q[ACDGHNPRSTY]|W.RE[DFHILNVY]GAQL[AGIRTV]R[ACDFGHILNPRSTVY][AG]DDL[ADHILNPTV][AEKT]Q[ACDGHNPRSTY]|W[DEHIKLMNQV].E[DFHILNVY]GAQL[AGIRTV]R[ACDFGHILNPRSTVY][AG]DDL[ADHILNPTV][AEKT]Q[ACDGHNPRSTY]|W[DEHIKLMNQV]R.[DFHILNVY]GAQL[AGIRTV]R[ACDFGHILNPRSTVY][AG]DDL[ADHILNPTV][AEKT]Q[ACDGHNPRSTY]|W[DEHIKLMNQV]RE.GAQL[AGIRTV]R[ACDFGHILNPRSTVY][AG]DDL[ADHILNPTV][AEKT]Q[ACDGHNPRSTY]|W[DEHIKLMNQV]RE[DFHILNVY].AQL[AGIRTV]R[ACDFGHILNPRSTVY][AG]DDL[ADHILNPTV][AEKT]Q[ACDGHNPRSTY]|W[DEHIKLMNQV]RE[DFHILNVY]G.QL[AGIRTV]R[ACDFGHILNPRSTVY][AG]DDL[ADHILNPTV][AEKT]Q[ACDGHNPRSTY]|W[DEHIKLMNQV]RE[DFHILNVY]GA.L[AGIRTV]R[ACDFGHILNPRSTVY][AG]DDL[ADHILNPTV][AEKT]Q[ACDGHNPRSTY]|W[DEHIKLMNQV]RE[DFHILNVY]GAQ.[AGIRTV]R[ACDFGHILNPRSTVY][AG]DDL[ADHILNPTV][AEKT]Q[ACDGHNPRSTY]|W[DEHIKLMNQV]RE[DFHILNVY]GAQL.R[ACDFGHILNPRSTVY][AG]DDL[ADHILNPTV][AEKT]Q[ACDGHNPRSTY]|W[DEHIKLMNQV]RE[DFHILNVY]GAQL[AGIRTV].[ACDFGHILNPRSTVY][AG]DDL[ADHILNPTV][AEKT]Q[ACDGHNPRSTY]|W[DEHIKLMNQV]RE[DFHILNVY]GAQL[AGIRTV]R.[AG]DDL[ADHILNPTV][AEKT]Q[ACDGHNPRSTY]|W[DEHIKLMNQV]RE[DFHILNVY]GAQL[AGIRTV]R[ACDFGHILNPRSTVY].DDL[ADHILNPTV][AEKT]Q[ACDGHNPRSTY]|W[DEHIKLMNQV]RE[DFHILNVY]GAQL[AGIRTV]R[ACDFGHILNPRSTVY][AG].DL[ADHILNPTV][AEKT]Q[ACDGHNPRSTY]|W[DEHIKLMNQV]RE[DFHILNVY]GAQL[AGIRTV]R[ACDFGHILNPRSTVY][AG]D.L[ADHILNPTV][AEKT]Q[ACDGHNPRSTY]|W[DEHIKLMNQV]RE[DFHILNVY]GAQL[AGIRTV]R[ACDFGHILNPRSTVY][AG]DD.[ADHILNPTV][AEKT]Q[ACDGHNPRSTY]|W[DEHIKLMNQV]RE[DFHILNVY]GAQL[AGIRTV]R[ACDFGHILNPRSTVY][AG]DDL.[AEKT]Q[ACDGHNPRSTY]|W[DEHIKLMNQV]RE[DFHILNVY]GAQL[AGIRTV]R[ACDFGHILNPRSTVY][AG]DDL[ADHILNPTV].Q[ACDGHNPRSTY]|W[DEHIKLMNQV]RE[DFHILNVY]GAQL[AGIRTV]R[ACDFGHILNPRSTVY][AG]DDL[ADHILNPTV][AEKT].[ACDGHNPRSTY]|W[DEHIKLMNQV]RE[DFHILNVY]GAQL[AGIRTV]R[ACDFGHILNPRSTVY][AG]DDL[ADHILNPTV][AEKT]Q."    
                                                        )]['count'].sum()))
Xlib_1mut_Run1C=np.array(Xlib_1mut_Run1C)

Xlib_1mut_RedoC=[]
for each in sortsRedo:
    Xlib_1mut_RedoC.append(float(each[each['seq'].str.contains("C")&each['seq'].str.contains(".[DEHIKLMNQV]RE[DFHILNVY]GAQL[AGIRTV]R[ACDFGHILNPRSTVY][AG]DDL[ADHILNPTV][AEKT]Q[ACDGHNPRSTY]|W.RE[DFHILNVY]GAQL[AGIRTV]R[ACDFGHILNPRSTVY][AG]DDL[ADHILNPTV][AEKT]Q[ACDGHNPRSTY]|W[DEHIKLMNQV].E[DFHILNVY]GAQL[AGIRTV]R[ACDFGHILNPRSTVY][AG]DDL[ADHILNPTV][AEKT]Q[ACDGHNPRSTY]|W[DEHIKLMNQV]R.[DFHILNVY]GAQL[AGIRTV]R[ACDFGHILNPRSTVY][AG]DDL[ADHILNPTV][AEKT]Q[ACDGHNPRSTY]|W[DEHIKLMNQV]RE.GAQL[AGIRTV]R[ACDFGHILNPRSTVY][AG]DDL[ADHILNPTV][AEKT]Q[ACDGHNPRSTY]|W[DEHIKLMNQV]RE[DFHILNVY].AQL[AGIRTV]R[ACDFGHILNPRSTVY][AG]DDL[ADHILNPTV][AEKT]Q[ACDGHNPRSTY]|W[DEHIKLMNQV]RE[DFHILNVY]G.QL[AGIRTV]R[ACDFGHILNPRSTVY][AG]DDL[ADHILNPTV][AEKT]Q[ACDGHNPRSTY]|W[DEHIKLMNQV]RE[DFHILNVY]GA.L[AGIRTV]R[ACDFGHILNPRSTVY][AG]DDL[ADHILNPTV][AEKT]Q[ACDGHNPRSTY]|W[DEHIKLMNQV]RE[DFHILNVY]GAQ.[AGIRTV]R[ACDFGHILNPRSTVY][AG]DDL[ADHILNPTV][AEKT]Q[ACDGHNPRSTY]|W[DEHIKLMNQV]RE[DFHILNVY]GAQL.R[ACDFGHILNPRSTVY][AG]DDL[ADHILNPTV][AEKT]Q[ACDGHNPRSTY]|W[DEHIKLMNQV]RE[DFHILNVY]GAQL[AGIRTV].[ACDFGHILNPRSTVY][AG]DDL[ADHILNPTV][AEKT]Q[ACDGHNPRSTY]|W[DEHIKLMNQV]RE[DFHILNVY]GAQL[AGIRTV]R.[AG]DDL[ADHILNPTV][AEKT]Q[ACDGHNPRSTY]|W[DEHIKLMNQV]RE[DFHILNVY]GAQL[AGIRTV]R[ACDFGHILNPRSTVY].DDL[ADHILNPTV][AEKT]Q[ACDGHNPRSTY]|W[DEHIKLMNQV]RE[DFHILNVY]GAQL[AGIRTV]R[ACDFGHILNPRSTVY][AG].DL[ADHILNPTV][AEKT]Q[ACDGHNPRSTY]|W[DEHIKLMNQV]RE[DFHILNVY]GAQL[AGIRTV]R[ACDFGHILNPRSTVY][AG]D.L[ADHILNPTV][AEKT]Q[ACDGHNPRSTY]|W[DEHIKLMNQV]RE[DFHILNVY]GAQL[AGIRTV]R[ACDFGHILNPRSTVY][AG]DD.[ADHILNPTV][AEKT]Q[ACDGHNPRSTY]|W[DEHIKLMNQV]RE[DFHILNVY]GAQL[AGIRTV]R[ACDFGHILNPRSTVY][AG]DDL.[AEKT]Q[ACDGHNPRSTY]|W[DEHIKLMNQV]RE[DFHILNVY]GAQL[AGIRTV]R[ACDFGHILNPRSTVY][AG]DDL[ADHILNPTV].Q[ACDGHNPRSTY]|W[DEHIKLMNQV]RE[DFHILNVY]GAQL[AGIRTV]R[ACDFGHILNPRSTVY][AG]DDL[ADHILNPTV][AEKT].[ACDGHNPRSTY]|W[DEHIKLMNQV]RE[DFHILNVY]GAQL[AGIRTV]R[ACDFGHILNPRSTVY][AG]DDL[ADHILNPTV][AEKT]Q."    
                                                        )]['count'].sum()))
Xlib_1mut_RedoC=np.array(Xlib_1mut_RedoC)

Mlib_1mut_redo=[]
for each in sortsRedo:
    Mlib_1mut_redo.append(float(each[each['seq'].str.contains(".A[A,C,D,F,G,H,L,P,R,S,V,Y][D,E,H,Q][A,I,T,V][A,G,I,S,T,V]AQL[A,E,G,I,K,R,T,V]RMADD[A,D,F,H,I,L,N,P,S,T,V,Y][D,E,H,K,N,Q]AQY|[A,G,P,R,S,T,W].[A,C,D,F,G,H,L,P,R,S,V,Y][D,E,H,Q][A,I,T,V][A,G,I,S,T,V]AQL[A,E,G,I,K,R,T,V]RMADD[A,D,F,H,I,L,N,P,S,T,V,Y][D,E,H,K,N,Q]AQY|[A,G,P,R,S,T,W]A.[D,E,H,Q][A,I,T,V][A,G,I,S,T,V]AQL[A,E,G,I,K,R,T,V]RMADD[A,D,F,H,I,L,N,P,S,T,V,Y][D,E,H,K,N,Q]AQY|[A,G,P,R,S,T,W]A[A,C,D,F,G,H,L,P,R,S,V,Y].[A,I,T,V][A,G,I,S,T,V]AQL[A,E,G,I,K,R,T,V]RMADD[A,D,F,H,I,L,N,P,S,T,V,Y][D,E,H,K,N,Q]AQY|[A,G,P,R,S,T,W]A[A,C,D,F,G,H,L,P,R,S,V,Y][D,E,H,Q].[A,G,I,S,T,V]AQL[A,E,G,I,K,R,T,V]RMADD[A,D,F,H,I,L,N,P,S,T,V,Y][D,E,H,K,N,Q]AQY|[A,G,P,R,S,T,W]A[A,C,D,F,G,H,L,P,R,S,V,Y][D,E,H,Q][A,I,T,V].AQL[A,E,G,I,K,R,T,V]RMADD[A,D,F,H,I,L,N,P,S,T,V,Y][D,E,H,K,N,Q]AQY|[A,G,P,R,S,T,W]A[A,C,D,F,G,H,L,P,R,S,V,Y][D,E,H,Q][A,I,T,V][A,G,I,S,T,V].QL[A,E,G,I,K,R,T,V]RMADD[A,D,F,H,I,L,N,P,S,T,V,Y][D,E,H,K,N,Q]AQY|[A,G,P,R,S,T,W]A[A,C,D,F,G,H,L,P,R,S,V,Y][D,E,H,Q][A,I,T,V][A,G,I,S,T,V]A.L[A,E,G,I,K,R,T,V]RMADD[A,D,F,H,I,L,N,P,S,T,V,Y][D,E,H,K,N,Q]AQY|[A,G,P,R,S,T,W]A[A,C,D,F,G,H,L,P,R,S,V,Y][D,E,H,Q][A,I,T,V][A,G,I,S,T,V]AQ.[A,E,G,I,K,R,T,V]RMADD[A,D,F,H,I,L,N,P,S,T,V,Y][D,E,H,K,N,Q]AQY|[A,G,P,R,S,T,W]A[A,C,D,F,G,H,L,P,R,S,V,Y][D,E,H,Q][A,I,T,V][A,G,I,S,T,V]AQL.RMADD[A,D,F,H,I,L,N,P,S,T,V,Y][D,E,H,K,N,Q]AQY|[A,G,P,R,S,T,W]A[A,C,D,F,G,H,L,P,R,S,V,Y][D,E,H,Q][A,I,T,V][A,G,I,S,T,V]AQL[A,E,G,I,K,R,T,V].MADD[A,D,F,H,I,L,N,P,S,T,V,Y][D,E,H,K,N,Q]AQY|[A,G,P,R,S,T,W]A[A,C,D,F,G,H,L,P,R,S,V,Y][D,E,H,Q][A,I,T,V][A,G,I,S,T,V]AQL[A,E,G,I,K,R,T,V]R.ADD[A,D,F,H,I,L,N,P,S,T,V,Y][D,E,H,K,N,Q]AQY|[A,G,P,R,S,T,W]A[A,C,D,F,G,H,L,P,R,S,V,Y][D,E,H,Q][A,I,T,V][A,G,I,S,T,V]AQL[A,E,G,I,K,R,T,V]RM.DD[A,D,F,H,I,L,N,P,S,T,V,Y][D,E,H,K,N,Q]AQY|[A,G,P,R,S,T,W]A[A,C,D,F,G,H,L,P,R,S,V,Y][D,E,H,Q][A,I,T,V][A,G,I,S,T,V]AQL[A,E,G,I,K,R,T,V]RMA.D[A,D,F,H,I,L,N,P,S,T,V,Y][D,E,H,K,N,Q]AQY|[A,G,P,R,S,T,W]A[A,C,D,F,G,H,L,P,R,S,V,Y][D,E,H,Q][A,I,T,V][A,G,I,S,T,V]AQL[A,E,G,I,K,R,T,V]RMAD.[A,D,F,H,I,L,N,P,S,T,V,Y][D,E,H,K,N,Q]AQY|[A,G,P,R,S,T,W]A[A,C,D,F,G,H,L,P,R,S,V,Y][D,E,H,Q][A,I,T,V][A,G,I,S,T,V]AQL[A,E,G,I,K,R,T,V]RMADD.[D,E,H,K,N,Q]AQY|[A,G,P,R,S,T,W]A[A,C,D,F,G,H,L,P,R,S,V,Y][D,E,H,Q][A,I,T,V][A,G,I,S,T,V]AQL[A,E,G,I,K,R,T,V]RMADD[A,D,F,H,I,L,N,P,S,T,V,Y].AQY|[A,G,P,R,S,T,W]A[A,C,D,F,G,H,L,P,R,S,V,Y][D,E,H,Q][A,I,T,V][A,G,I,S,T,V]AQL[A,E,G,I,K,R,T,V]RMADD[A,D,F,H,I,L,N,P,S,T,V,Y][D,E,H,K,N,Q].QY|[A,G,P,R,S,T,W]A[A,C,D,F,G,H,L,P,R,S,V,Y][D,E,H,Q][A,I,T,V][A,G,I,S,T,V]AQL[A,E,G,I,K,R,T,V]RMADD[A,D,F,H,I,L,N,P,S,T,V,Y][D,E,H,K,N,Q]A.Y|[A,G,P,R,S,T,W]A[A,C,D,F,G,H,L,P,R,S,V,Y][D,E,H,Q][A,I,T,V][A,G,I,S,T,V]AQL[A,E,G,I,K,R,T,V]RMADD[A,D,F,H,I,L,N,P,S,T,V,Y][D,E,H,K,N,Q]AQ."
                                                        )]['count'].sum()))
Mlib_1mut_redo=np.array(Mlib_1mut_redo)

Mlib_1mut_Run1=[]
for each in sorts:
    Mlib_1mut_Run1.append(float(each[each['seq'].str.contains(".A[A,C,D,F,G,H,L,P,R,S,V,Y][D,E,H,Q][A,I,T,V][A,G,I,S,T,V]AQL[A,E,G,I,K,R,T,V]RMADD[A,D,F,H,I,L,N,P,S,T,V,Y][D,E,H,K,N,Q]AQY|[A,G,P,R,S,T,W].[A,C,D,F,G,H,L,P,R,S,V,Y][D,E,H,Q][A,I,T,V][A,G,I,S,T,V]AQL[A,E,G,I,K,R,T,V]RMADD[A,D,F,H,I,L,N,P,S,T,V,Y][D,E,H,K,N,Q]AQY|[A,G,P,R,S,T,W]A.[D,E,H,Q][A,I,T,V][A,G,I,S,T,V]AQL[A,E,G,I,K,R,T,V]RMADD[A,D,F,H,I,L,N,P,S,T,V,Y][D,E,H,K,N,Q]AQY|[A,G,P,R,S,T,W]A[A,C,D,F,G,H,L,P,R,S,V,Y].[A,I,T,V][A,G,I,S,T,V]AQL[A,E,G,I,K,R,T,V]RMADD[A,D,F,H,I,L,N,P,S,T,V,Y][D,E,H,K,N,Q]AQY|[A,G,P,R,S,T,W]A[A,C,D,F,G,H,L,P,R,S,V,Y][D,E,H,Q].[A,G,I,S,T,V]AQL[A,E,G,I,K,R,T,V]RMADD[A,D,F,H,I,L,N,P,S,T,V,Y][D,E,H,K,N,Q]AQY|[A,G,P,R,S,T,W]A[A,C,D,F,G,H,L,P,R,S,V,Y][D,E,H,Q][A,I,T,V].AQL[A,E,G,I,K,R,T,V]RMADD[A,D,F,H,I,L,N,P,S,T,V,Y][D,E,H,K,N,Q]AQY|[A,G,P,R,S,T,W]A[A,C,D,F,G,H,L,P,R,S,V,Y][D,E,H,Q][A,I,T,V][A,G,I,S,T,V].QL[A,E,G,I,K,R,T,V]RMADD[A,D,F,H,I,L,N,P,S,T,V,Y][D,E,H,K,N,Q]AQY|[A,G,P,R,S,T,W]A[A,C,D,F,G,H,L,P,R,S,V,Y][D,E,H,Q][A,I,T,V][A,G,I,S,T,V]A.L[A,E,G,I,K,R,T,V]RMADD[A,D,F,H,I,L,N,P,S,T,V,Y][D,E,H,K,N,Q]AQY|[A,G,P,R,S,T,W]A[A,C,D,F,G,H,L,P,R,S,V,Y][D,E,H,Q][A,I,T,V][A,G,I,S,T,V]AQ.[A,E,G,I,K,R,T,V]RMADD[A,D,F,H,I,L,N,P,S,T,V,Y][D,E,H,K,N,Q]AQY|[A,G,P,R,S,T,W]A[A,C,D,F,G,H,L,P,R,S,V,Y][D,E,H,Q][A,I,T,V][A,G,I,S,T,V]AQL.RMADD[A,D,F,H,I,L,N,P,S,T,V,Y][D,E,H,K,N,Q]AQY|[A,G,P,R,S,T,W]A[A,C,D,F,G,H,L,P,R,S,V,Y][D,E,H,Q][A,I,T,V][A,G,I,S,T,V]AQL[A,E,G,I,K,R,T,V].MADD[A,D,F,H,I,L,N,P,S,T,V,Y][D,E,H,K,N,Q]AQY|[A,G,P,R,S,T,W]A[A,C,D,F,G,H,L,P,R,S,V,Y][D,E,H,Q][A,I,T,V][A,G,I,S,T,V]AQL[A,E,G,I,K,R,T,V]R.ADD[A,D,F,H,I,L,N,P,S,T,V,Y][D,E,H,K,N,Q]AQY|[A,G,P,R,S,T,W]A[A,C,D,F,G,H,L,P,R,S,V,Y][D,E,H,Q][A,I,T,V][A,G,I,S,T,V]AQL[A,E,G,I,K,R,T,V]RM.DD[A,D,F,H,I,L,N,P,S,T,V,Y][D,E,H,K,N,Q]AQY|[A,G,P,R,S,T,W]A[A,C,D,F,G,H,L,P,R,S,V,Y][D,E,H,Q][A,I,T,V][A,G,I,S,T,V]AQL[A,E,G,I,K,R,T,V]RMA.D[A,D,F,H,I,L,N,P,S,T,V,Y][D,E,H,K,N,Q]AQY|[A,G,P,R,S,T,W]A[A,C,D,F,G,H,L,P,R,S,V,Y][D,E,H,Q][A,I,T,V][A,G,I,S,T,V]AQL[A,E,G,I,K,R,T,V]RMAD.[A,D,F,H,I,L,N,P,S,T,V,Y][D,E,H,K,N,Q]AQY|[A,G,P,R,S,T,W]A[A,C,D,F,G,H,L,P,R,S,V,Y][D,E,H,Q][A,I,T,V][A,G,I,S,T,V]AQL[A,E,G,I,K,R,T,V]RMADD.[D,E,H,K,N,Q]AQY|[A,G,P,R,S,T,W]A[A,C,D,F,G,H,L,P,R,S,V,Y][D,E,H,Q][A,I,T,V][A,G,I,S,T,V]AQL[A,E,G,I,K,R,T,V]RMADD[A,D,F,H,I,L,N,P,S,T,V,Y].AQY|[A,G,P,R,S,T,W]A[A,C,D,F,G,H,L,P,R,S,V,Y][D,E,H,Q][A,I,T,V][A,G,I,S,T,V]AQL[A,E,G,I,K,R,T,V]RMADD[A,D,F,H,I,L,N,P,S,T,V,Y][D,E,H,K,N,Q].QY|[A,G,P,R,S,T,W]A[A,C,D,F,G,H,L,P,R,S,V,Y][D,E,H,Q][A,I,T,V][A,G,I,S,T,V]AQL[A,E,G,I,K,R,T,V]RMADD[A,D,F,H,I,L,N,P,S,T,V,Y][D,E,H,K,N,Q]A.Y|[A,G,P,R,S,T,W]A[A,C,D,F,G,H,L,P,R,S,V,Y][D,E,H,Q][A,I,T,V][A,G,I,S,T,V]AQL[A,E,G,I,K,R,T,V]RMADD[A,D,F,H,I,L,N,P,S,T,V,Y][D,E,H,K,N,Q]AQ."
                                                        )]['count'].sum()))
Mlib_1mut_Run1=np.array(Mlib_1mut_Run1)

Mlib_1mut_Run1C=[]
for each in sorts:
    Mlib_1mut_Run1C.append(float(each[each['seq'].str.contains("C")&each['seq'].str.contains(".A[A,C,D,F,G,H,L,P,R,S,V,Y][D,E,H,Q][A,I,T,V][A,G,I,S,T,V]AQL[A,E,G,I,K,R,T,V]RMADD[A,D,F,H,I,L,N,P,S,T,V,Y][D,E,H,K,N,Q]AQY|[A,G,P,R,S,T,W].[A,C,D,F,G,H,L,P,R,S,V,Y][D,E,H,Q][A,I,T,V][A,G,I,S,T,V]AQL[A,E,G,I,K,R,T,V]RMADD[A,D,F,H,I,L,N,P,S,T,V,Y][D,E,H,K,N,Q]AQY|[A,G,P,R,S,T,W]A.[D,E,H,Q][A,I,T,V][A,G,I,S,T,V]AQL[A,E,G,I,K,R,T,V]RMADD[A,D,F,H,I,L,N,P,S,T,V,Y][D,E,H,K,N,Q]AQY|[A,G,P,R,S,T,W]A[A,C,D,F,G,H,L,P,R,S,V,Y].[A,I,T,V][A,G,I,S,T,V]AQL[A,E,G,I,K,R,T,V]RMADD[A,D,F,H,I,L,N,P,S,T,V,Y][D,E,H,K,N,Q]AQY|[A,G,P,R,S,T,W]A[A,C,D,F,G,H,L,P,R,S,V,Y][D,E,H,Q].[A,G,I,S,T,V]AQL[A,E,G,I,K,R,T,V]RMADD[A,D,F,H,I,L,N,P,S,T,V,Y][D,E,H,K,N,Q]AQY|[A,G,P,R,S,T,W]A[A,C,D,F,G,H,L,P,R,S,V,Y][D,E,H,Q][A,I,T,V].AQL[A,E,G,I,K,R,T,V]RMADD[A,D,F,H,I,L,N,P,S,T,V,Y][D,E,H,K,N,Q]AQY|[A,G,P,R,S,T,W]A[A,C,D,F,G,H,L,P,R,S,V,Y][D,E,H,Q][A,I,T,V][A,G,I,S,T,V].QL[A,E,G,I,K,R,T,V]RMADD[A,D,F,H,I,L,N,P,S,T,V,Y][D,E,H,K,N,Q]AQY|[A,G,P,R,S,T,W]A[A,C,D,F,G,H,L,P,R,S,V,Y][D,E,H,Q][A,I,T,V][A,G,I,S,T,V]A.L[A,E,G,I,K,R,T,V]RMADD[A,D,F,H,I,L,N,P,S,T,V,Y][D,E,H,K,N,Q]AQY|[A,G,P,R,S,T,W]A[A,C,D,F,G,H,L,P,R,S,V,Y][D,E,H,Q][A,I,T,V][A,G,I,S,T,V]AQ.[A,E,G,I,K,R,T,V]RMADD[A,D,F,H,I,L,N,P,S,T,V,Y][D,E,H,K,N,Q]AQY|[A,G,P,R,S,T,W]A[A,C,D,F,G,H,L,P,R,S,V,Y][D,E,H,Q][A,I,T,V][A,G,I,S,T,V]AQL.RMADD[A,D,F,H,I,L,N,P,S,T,V,Y][D,E,H,K,N,Q]AQY|[A,G,P,R,S,T,W]A[A,C,D,F,G,H,L,P,R,S,V,Y][D,E,H,Q][A,I,T,V][A,G,I,S,T,V]AQL[A,E,G,I,K,R,T,V].MADD[A,D,F,H,I,L,N,P,S,T,V,Y][D,E,H,K,N,Q]AQY|[A,G,P,R,S,T,W]A[A,C,D,F,G,H,L,P,R,S,V,Y][D,E,H,Q][A,I,T,V][A,G,I,S,T,V]AQL[A,E,G,I,K,R,T,V]R.ADD[A,D,F,H,I,L,N,P,S,T,V,Y][D,E,H,K,N,Q]AQY|[A,G,P,R,S,T,W]A[A,C,D,F,G,H,L,P,R,S,V,Y][D,E,H,Q][A,I,T,V][A,G,I,S,T,V]AQL[A,E,G,I,K,R,T,V]RM.DD[A,D,F,H,I,L,N,P,S,T,V,Y][D,E,H,K,N,Q]AQY|[A,G,P,R,S,T,W]A[A,C,D,F,G,H,L,P,R,S,V,Y][D,E,H,Q][A,I,T,V][A,G,I,S,T,V]AQL[A,E,G,I,K,R,T,V]RMA.D[A,D,F,H,I,L,N,P,S,T,V,Y][D,E,H,K,N,Q]AQY|[A,G,P,R,S,T,W]A[A,C,D,F,G,H,L,P,R,S,V,Y][D,E,H,Q][A,I,T,V][A,G,I,S,T,V]AQL[A,E,G,I,K,R,T,V]RMAD.[A,D,F,H,I,L,N,P,S,T,V,Y][D,E,H,K,N,Q]AQY|[A,G,P,R,S,T,W]A[A,C,D,F,G,H,L,P,R,S,V,Y][D,E,H,Q][A,I,T,V][A,G,I,S,T,V]AQL[A,E,G,I,K,R,T,V]RMADD.[D,E,H,K,N,Q]AQY|[A,G,P,R,S,T,W]A[A,C,D,F,G,H,L,P,R,S,V,Y][D,E,H,Q][A,I,T,V][A,G,I,S,T,V]AQL[A,E,G,I,K,R,T,V]RMADD[A,D,F,H,I,L,N,P,S,T,V,Y].AQY|[A,G,P,R,S,T,W]A[A,C,D,F,G,H,L,P,R,S,V,Y][D,E,H,Q][A,I,T,V][A,G,I,S,T,V]AQL[A,E,G,I,K,R,T,V]RMADD[A,D,F,H,I,L,N,P,S,T,V,Y][D,E,H,K,N,Q].QY|[A,G,P,R,S,T,W]A[A,C,D,F,G,H,L,P,R,S,V,Y][D,E,H,Q][A,I,T,V][A,G,I,S,T,V]AQL[A,E,G,I,K,R,T,V]RMADD[A,D,F,H,I,L,N,P,S,T,V,Y][D,E,H,K,N,Q]A.Y|[A,G,P,R,S,T,W]A[A,C,D,F,G,H,L,P,R,S,V,Y][D,E,H,Q][A,I,T,V][A,G,I,S,T,V]AQL[A,E,G,I,K,R,T,V]RMADD[A,D,F,H,I,L,N,P,S,T,V,Y][D,E,H,K,N,Q]AQ."
                                                        )]['count'].sum()))
Mlib_1mut_Run1C=np.array(Mlib_1mut_Run1C)

Mlib_1mut_RedoC=[]
for each in sortsRedo:
    Mlib_1mut_RedoC.append(float(each[each['seq'].str.contains("C")&each['seq'].str.contains(".A[A,C,D,F,G,H,L,P,R,S,V,Y][D,E,H,Q][A,I,T,V][A,G,I,S,T,V]AQL[A,E,G,I,K,R,T,V]RMADD[A,D,F,H,I,L,N,P,S,T,V,Y][D,E,H,K,N,Q]AQY|[A,G,P,R,S,T,W].[A,C,D,F,G,H,L,P,R,S,V,Y][D,E,H,Q][A,I,T,V][A,G,I,S,T,V]AQL[A,E,G,I,K,R,T,V]RMADD[A,D,F,H,I,L,N,P,S,T,V,Y][D,E,H,K,N,Q]AQY|[A,G,P,R,S,T,W]A.[D,E,H,Q][A,I,T,V][A,G,I,S,T,V]AQL[A,E,G,I,K,R,T,V]RMADD[A,D,F,H,I,L,N,P,S,T,V,Y][D,E,H,K,N,Q]AQY|[A,G,P,R,S,T,W]A[A,C,D,F,G,H,L,P,R,S,V,Y].[A,I,T,V][A,G,I,S,T,V]AQL[A,E,G,I,K,R,T,V]RMADD[A,D,F,H,I,L,N,P,S,T,V,Y][D,E,H,K,N,Q]AQY|[A,G,P,R,S,T,W]A[A,C,D,F,G,H,L,P,R,S,V,Y][D,E,H,Q].[A,G,I,S,T,V]AQL[A,E,G,I,K,R,T,V]RMADD[A,D,F,H,I,L,N,P,S,T,V,Y][D,E,H,K,N,Q]AQY|[A,G,P,R,S,T,W]A[A,C,D,F,G,H,L,P,R,S,V,Y][D,E,H,Q][A,I,T,V].AQL[A,E,G,I,K,R,T,V]RMADD[A,D,F,H,I,L,N,P,S,T,V,Y][D,E,H,K,N,Q]AQY|[A,G,P,R,S,T,W]A[A,C,D,F,G,H,L,P,R,S,V,Y][D,E,H,Q][A,I,T,V][A,G,I,S,T,V].QL[A,E,G,I,K,R,T,V]RMADD[A,D,F,H,I,L,N,P,S,T,V,Y][D,E,H,K,N,Q]AQY|[A,G,P,R,S,T,W]A[A,C,D,F,G,H,L,P,R,S,V,Y][D,E,H,Q][A,I,T,V][A,G,I,S,T,V]A.L[A,E,G,I,K,R,T,V]RMADD[A,D,F,H,I,L,N,P,S,T,V,Y][D,E,H,K,N,Q]AQY|[A,G,P,R,S,T,W]A[A,C,D,F,G,H,L,P,R,S,V,Y][D,E,H,Q][A,I,T,V][A,G,I,S,T,V]AQ.[A,E,G,I,K,R,T,V]RMADD[A,D,F,H,I,L,N,P,S,T,V,Y][D,E,H,K,N,Q]AQY|[A,G,P,R,S,T,W]A[A,C,D,F,G,H,L,P,R,S,V,Y][D,E,H,Q][A,I,T,V][A,G,I,S,T,V]AQL.RMADD[A,D,F,H,I,L,N,P,S,T,V,Y][D,E,H,K,N,Q]AQY|[A,G,P,R,S,T,W]A[A,C,D,F,G,H,L,P,R,S,V,Y][D,E,H,Q][A,I,T,V][A,G,I,S,T,V]AQL[A,E,G,I,K,R,T,V].MADD[A,D,F,H,I,L,N,P,S,T,V,Y][D,E,H,K,N,Q]AQY|[A,G,P,R,S,T,W]A[A,C,D,F,G,H,L,P,R,S,V,Y][D,E,H,Q][A,I,T,V][A,G,I,S,T,V]AQL[A,E,G,I,K,R,T,V]R.ADD[A,D,F,H,I,L,N,P,S,T,V,Y][D,E,H,K,N,Q]AQY|[A,G,P,R,S,T,W]A[A,C,D,F,G,H,L,P,R,S,V,Y][D,E,H,Q][A,I,T,V][A,G,I,S,T,V]AQL[A,E,G,I,K,R,T,V]RM.DD[A,D,F,H,I,L,N,P,S,T,V,Y][D,E,H,K,N,Q]AQY|[A,G,P,R,S,T,W]A[A,C,D,F,G,H,L,P,R,S,V,Y][D,E,H,Q][A,I,T,V][A,G,I,S,T,V]AQL[A,E,G,I,K,R,T,V]RMA.D[A,D,F,H,I,L,N,P,S,T,V,Y][D,E,H,K,N,Q]AQY|[A,G,P,R,S,T,W]A[A,C,D,F,G,H,L,P,R,S,V,Y][D,E,H,Q][A,I,T,V][A,G,I,S,T,V]AQL[A,E,G,I,K,R,T,V]RMAD.[A,D,F,H,I,L,N,P,S,T,V,Y][D,E,H,K,N,Q]AQY|[A,G,P,R,S,T,W]A[A,C,D,F,G,H,L,P,R,S,V,Y][D,E,H,Q][A,I,T,V][A,G,I,S,T,V]AQL[A,E,G,I,K,R,T,V]RMADD.[D,E,H,K,N,Q]AQY|[A,G,P,R,S,T,W]A[A,C,D,F,G,H,L,P,R,S,V,Y][D,E,H,Q][A,I,T,V][A,G,I,S,T,V]AQL[A,E,G,I,K,R,T,V]RMADD[A,D,F,H,I,L,N,P,S,T,V,Y].AQY|[A,G,P,R,S,T,W]A[A,C,D,F,G,H,L,P,R,S,V,Y][D,E,H,Q][A,I,T,V][A,G,I,S,T,V]AQL[A,E,G,I,K,R,T,V]RMADD[A,D,F,H,I,L,N,P,S,T,V,Y][D,E,H,K,N,Q].QY|[A,G,P,R,S,T,W]A[A,C,D,F,G,H,L,P,R,S,V,Y][D,E,H,Q][A,I,T,V][A,G,I,S,T,V]AQL[A,E,G,I,K,R,T,V]RMADD[A,D,F,H,I,L,N,P,S,T,V,Y][D,E,H,K,N,Q]A.Y|[A,G,P,R,S,T,W]A[A,C,D,F,G,H,L,P,R,S,V,Y][D,E,H,Q][A,I,T,V][A,G,I,S,T,V]AQL[A,E,G,I,K,R,T,V]RMADD[A,D,F,H,I,L,N,P,S,T,V,Y][D,E,H,K,N,Q]AQ."
                                                        )]['count'].sum()))
Mlib_1mut_RedoC=np.array(Mlib_1mut_RedoC)

Cys_run1=[]
for each in sorts:
    Cys_run1.append(float(each[each['seq'].str.contains("C")]['count'].sum()))
Cys_run1=np.array(Cys_run1)

Cys_redo=[]
for each in sortsRedo:
    Cys_redo.append(float(each[each['seq'].str.contains("C")]['count'].sum()))
Cys_redo=np.array(Cys_redo)
```

In [17]:

```
#figure 3a
f, axes = plt.subplots(1, 1,figsize=(8, 4))
plt.xticks(np.arange(0, 4.1, 1.0))
a=axes.get_xticks().tolist()
a[:]=['initial','3','4','5$^\prime$','6$^\prime$']
axes.set_xticklabels(a)
Xlib_1=((Xlib_1mut_redo-Xlib_1mut_RedoC)/(totreads_redo-Cys_redo))#fraction of DNA reads that that don't encode for cysteine from Bcl-xl library. 
Mlib_1=((Mlib_1mut_redo-Mlib_1mut_RedoC)/(totreads_redo-Cys_redo))#fraction of DNA reads that that don't encode for cysteine from Mcl-1 library.
Flib_1=((Flib_1mut_redo-Flib_1mut_RedoC)/(totreads_redo-Cys_redo))#fraction of DNA reads that that don't encode for cysteine from Bfl-1 library.
Other_1=[1.,1.,1.,1.,1.]-Xlib_1-Mlib_1-Flib_1#fraction of DNA reads > 1 mutation away from Bcl-xl, Mcl-1, or Bfl-1 library
x=np.array([0.,1.,2.,3.,4.])
plt.stackplot(x, Flib_1,Mlib_1,Xlib_1,Other_1,colors=['#4878cf','#6acc65','#d65f5f','#b47cc7'])
plt.ylim((0, 1))
plt.ylabel('fraction of reads', size=24)
plt.xlabel('sort pool', size=24)
axes.tick_params(axis='x', labelsize=18)
axes.tick_params(axis='y', labelsize=18)
sns.despine()
```

In [18]:

```
#Assign unique sequences to Bcl-xl, Mcl-1, or Bfl-1 library. Allows for up to one amino acid mutation from theoretical library. 
compRedo4noC=compRedo4[~compRedo4['seq'].str.contains("C")]#filter out sequences containing cysteine
compRedo4noCnoB=compRedo4noC[compRedo4noC.bg=='P']#only consider sequences that are mutants of PUMA BH3
compRedo4noCnoB=compRedo4noCnoB.reset_index(drop=True)

LibcompRedo4noCnoB=compRedo4noCnoB
LibcompRedo4noCnoB['rank'] = LibcompRedo4noCnoB['count'].rank(ascending=0)
LibcompRedo4noCnoB['lib'] = 'o'

LibcompRedo4noCnoB.loc[LibcompRedo4noCnoB['seq'].str.contains(".[DEHIKLMNQV]RE[DFHILNVY]GAQL[AGIRTV]R[ACDFGHILNPRSTVY][AG]DDL[ADHILNPTV][AEKT]Q[ACDGHNPRSTY]|W.RE[DFHILNVY]GAQL[AGIRTV]R[ACDFGHILNPRSTVY][AG]DDL[ADHILNPTV][AEKT]Q[ACDGHNPRSTY]|W[DEHIKLMNQV].E[DFHILNVY]GAQL[AGIRTV]R[ACDFGHILNPRSTVY][AG]DDL[ADHILNPTV][AEKT]Q[ACDGHNPRSTY]|W[DEHIKLMNQV]R.[DFHILNVY]GAQL[AGIRTV]R[ACDFGHILNPRSTVY][AG]DDL[ADHILNPTV][AEKT]Q[ACDGHNPRSTY]|W[DEHIKLMNQV]RE.GAQL[AGIRTV]R[ACDFGHILNPRSTVY][AG]DDL[ADHILNPTV][AEKT]Q[ACDGHNPRSTY]|W[DEHIKLMNQV]RE[DFHILNVY].AQL[AGIRTV]R[ACDFGHILNPRSTVY][AG]DDL[ADHILNPTV][AEKT]Q[ACDGHNPRSTY]|W[DEHIKLMNQV]RE[DFHILNVY]G.QL[AGIRTV]R[ACDFGHILNPRSTVY][AG]DDL[ADHILNPTV][AEKT]Q[ACDGHNPRSTY]|W[DEHIKLMNQV]RE[DFHILNVY]GA.L[AGIRTV]R[ACDFGHILNPRSTVY][AG]DDL[ADHILNPTV][AEKT]Q[ACDGHNPRSTY]|W[DEHIKLMNQV]RE[DFHILNVY]GAQ.[AGIRTV]R[ACDFGHILNPRSTVY][AG]DDL[ADHILNPTV][AEKT]Q[ACDGHNPRSTY]|W[DEHIKLMNQV]RE[DFHILNVY]GAQL.R[ACDFGHILNPRSTVY][AG]DDL[ADHILNPTV][AEKT]Q[ACDGHNPRSTY]|W[DEHIKLMNQV]RE[DFHILNVY]GAQL[AGIRTV].[ACDFGHILNPRSTVY][AG]DDL[ADHILNPTV][AEKT]Q[ACDGHNPRSTY]|W[DEHIKLMNQV]RE[DFHILNVY]GAQL[AGIRTV]R.[AG]DDL[ADHILNPTV][AEKT]Q[ACDGHNPRSTY]|W[DEHIKLMNQV]RE[DFHILNVY]GAQL[AGIRTV]R[ACDFGHILNPRSTVY].DDL[ADHILNPTV][AEKT]Q[ACDGHNPRSTY]|W[DEHIKLMNQV]RE[DFHILNVY]GAQL[AGIRTV]R[ACDFGHILNPRSTVY][AG].DL[ADHILNPTV][AEKT]Q[ACDGHNPRSTY]|W[DEHIKLMNQV]RE[DFHILNVY]GAQL[AGIRTV]R[ACDFGHILNPRSTVY][AG]D.L[ADHILNPTV][AEKT]Q[ACDGHNPRSTY]|W[DEHIKLMNQV]RE[DFHILNVY]GAQL[AGIRTV]R[ACDFGHILNPRSTVY][AG]DD.[ADHILNPTV][AEKT]Q[ACDGHNPRSTY]|W[DEHIKLMNQV]RE[DFHILNVY]GAQL[AGIRTV]R[ACDFGHILNPRSTVY][AG]DDL.[AEKT]Q[ACDGHNPRSTY]|W[DEHIKLMNQV]RE[DFHILNVY]GAQL[AGIRTV]R[ACDFGHILNPRSTVY][AG]DDL[ADHILNPTV].Q[ACDGHNPRSTY]|W[DEHIKLMNQV]RE[DFHILNVY]GAQL[AGIRTV]R[ACDFGHILNPRSTVY][AG]DDL[ADHILNPTV][AEKT].[ACDGHNPRSTY]|W[DEHIKLMNQV]RE[DFHILNVY]GAQL[AGIRTV]R[ACDFGHILNPRSTVY][AG]DDL[ADHILNPTV][AEKT]Q."),"lib"]='x'
LibcompRedo4noCnoB.loc[LibcompRedo4noCnoB['seq'].str.contains(".A[A,C,D,F,G,H,L,P,R,S,V,Y][D,E,H,Q][A,I,T,V][A,G,I,S,T,V]AQL[A,E,G,I,K,R,T,V]RMADD[A,D,F,H,I,L,N,P,S,T,V,Y][D,E,H,K,N,Q]AQY|[A,G,P,R,S,T,W].[A,C,D,F,G,H,L,P,R,S,V,Y][D,E,H,Q][A,I,T,V][A,G,I,S,T,V]AQL[A,E,G,I,K,R,T,V]RMADD[A,D,F,H,I,L,N,P,S,T,V,Y][D,E,H,K,N,Q]AQY|[A,G,P,R,S,T,W]A.[D,E,H,Q][A,I,T,V][A,G,I,S,T,V]AQL[A,E,G,I,K,R,T,V]RMADD[A,D,F,H,I,L,N,P,S,T,V,Y][D,E,H,K,N,Q]AQY|[A,G,P,R,S,T,W]A[A,C,D,F,G,H,L,P,R,S,V,Y].[A,I,T,V][A,G,I,S,T,V]AQL[A,E,G,I,K,R,T,V]RMADD[A,D,F,H,I,L,N,P,S,T,V,Y][D,E,H,K,N,Q]AQY|[A,G,P,R,S,T,W]A[A,C,D,F,G,H,L,P,R,S,V,Y][D,E,H,Q].[A,G,I,S,T,V]AQL[A,E,G,I,K,R,T,V]RMADD[A,D,F,H,I,L,N,P,S,T,V,Y][D,E,H,K,N,Q]AQY|[A,G,P,R,S,T,W]A[A,C,D,F,G,H,L,P,R,S,V,Y][D,E,H,Q][A,I,T,V].AQL[A,E,G,I,K,R,T,V]RMADD[A,D,F,H,I,L,N,P,S,T,V,Y][D,E,H,K,N,Q]AQY|[A,G,P,R,S,T,W]A[A,C,D,F,G,H,L,P,R,S,V,Y][D,E,H,Q][A,I,T,V][A,G,I,S,T,V].QL[A,E,G,I,K,R,T,V]RMADD[A,D,F,H,I,L,N,P,S,T,V,Y][D,E,H,K,N,Q]AQY|[A,G,P,R,S,T,W]A[A,C,D,F,G,H,L,P,R,S,V,Y][D,E,H,Q][A,I,T,V][A,G,I,S,T,V]A.L[A,E,G,I,K,R,T,V]RMADD[A,D,F,H,I,L,N,P,S,T,V,Y][D,E,H,K,N,Q]AQY|[A,G,P,R,S,T,W]A[A,C,D,F,G,H,L,P,R,S,V,Y][D,E,H,Q][A,I,T,V][A,G,I,S,T,V]AQ.[A,E,G,I,K,R,T,V]RMADD[A,D,F,H,I,L,N,P,S,T,V,Y][D,E,H,K,N,Q]AQY|[A,G,P,R,S,T,W]A[A,C,D,F,G,H,L,P,R,S,V,Y][D,E,H,Q][A,I,T,V][A,G,I,S,T,V]AQL.RMADD[A,D,F,H,I,L,N,P,S,T,V,Y][D,E,H,K,N,Q]AQY|[A,G,P,R,S,T,W]A[A,C,D,F,G,H,L,P,R,S,V,Y][D,E,H,Q][A,I,T,V][A,G,I,S,T,V]AQL[A,E,G,I,K,R,T,V].MADD[A,D,F,H,I,L,N,P,S,T,V,Y][D,E,H,K,N,Q]AQY|[A,G,P,R,S,T,W]A[A,C,D,F,G,H,L,P,R,S,V,Y][D,E,H,Q][A,I,T,V][A,G,I,S,T,V]AQL[A,E,G,I,K,R,T,V]R.ADD[A,D,F,H,I,L,N,P,S,T,V,Y][D,E,H,K,N,Q]AQY|[A,G,P,R,S,T,W]A[A,C,D,F,G,H,L,P,R,S,V,Y][D,E,H,Q][A,I,T,V][A,G,I,S,T,V]AQL[A,E,G,I,K,R,T,V]RM.DD[A,D,F,H,I,L,N,P,S,T,V,Y][D,E,H,K,N,Q]AQY|[A,G,P,R,S,T,W]A[A,C,D,F,G,H,L,P,R,S,V,Y][D,E,H,Q][A,I,T,V][A,G,I,S,T,V]AQL[A,E,G,I,K,R,T,V]RMA.D[A,D,F,H,I,L,N,P,S,T,V,Y][D,E,H,K,N,Q]AQY|[A,G,P,R,S,T,W]A[A,C,D,F,G,H,L,P,R,S,V,Y][D,E,H,Q][A,I,T,V][A,G,I,S,T,V]AQL[A,E,G,I,K,R,T,V]RMAD.[A,D,F,H,I,L,N,P,S,T,V,Y][D,E,H,K,N,Q]AQY|[A,G,P,R,S,T,W]A[A,C,D,F,G,H,L,P,R,S,V,Y][D,E,H,Q][A,I,T,V][A,G,I,S,T,V]AQL[A,E,G,I,K,R,T,V]RMADD.[D,E,H,K,N,Q]AQY|[A,G,P,R,S,T,W]A[A,C,D,F,G,H,L,P,R,S,V,Y][D,E,H,Q][A,I,T,V][A,G,I,S,T,V]AQL[A,E,G,I,K,R,T,V]RMADD[A,D,F,H,I,L,N,P,S,T,V,Y].AQY|[A,G,P,R,S,T,W]A[A,C,D,F,G,H,L,P,R,S,V,Y][D,E,H,Q][A,I,T,V][A,G,I,S,T,V]AQL[A,E,G,I,K,R,T,V]RMADD[A,D,F,H,I,L,N,P,S,T,V,Y][D,E,H,K,N,Q].QY|[A,G,P,R,S,T,W]A[A,C,D,F,G,H,L,P,R,S,V,Y][D,E,H,Q][A,I,T,V][A,G,I,S,T,V]AQL[A,E,G,I,K,R,T,V]RMADD[A,D,F,H,I,L,N,P,S,T,V,Y][D,E,H,K,N,Q]A.Y|[A,G,P,R,S,T,W]A[A,C,D,F,G,H,L,P,R,S,V,Y][D,E,H,Q][A,I,T,V][A,G,I,S,T,V]AQL[A,E,G,I,K,R,T,V]RMADD[A,D,F,H,I,L,N,P,S,T,V,Y][D,E,H,K,N,Q]AQ."),"lib"]='m'
LibcompRedo4noCnoB.loc[LibcompRedo4noCnoB['seq'].str.contains(".[A,E,I,K,L,P,Q,T,V]REI[A,C,D,G,S,Y]A[C,D,F,G,H,I,L,N,R,S,V,Y]LRR[A,C,F,G,I,L,P,R,S,T,V,]AD[D,E,H,I,K,L,M,N,Q,V][A,D,F,H,I,L,N,P,S,T,V,Y]NAQ[A,F,I,L,P,S,T,V]|W.REI[A,C,D,G,S,Y]A[C,D,F,G,H,I,L,N,R,S,V,Y]LRR[A,C,F,G,I,L,P,R,S,T,V,]AD[D,E,H,I,K,L,M,N,Q,V][A,D,F,H,I,L,N,P,S,T,V,Y]NAQ[A,F,I,L,P,S,T,V]|W[A,E,I,K,L,P,Q,T,V].EI[A,C,D,G,S,Y]A[C,D,F,G,H,I,L,N,R,S,V,Y]LRR[A,C,F,G,I,L,P,R,S,T,V,]AD[D,E,H,I,K,L,M,N,Q,V][A,D,F,H,I,L,N,P,S,T,V,Y]NAQ[A,F,I,L,P,S,T,V]|W[A,E,I,K,L,P,Q,T,V]R.I[A,C,D,G,S,Y]A[C,D,F,G,H,I,L,N,R,S,V,Y]LRR[A,C,F,G,I,L,P,R,S,T,V,]AD[D,E,H,I,K,L,M,N,Q,V][A,D,F,H,I,L,N,P,S,T,V,Y]NAQ[A,F,I,L,P,S,T,V]|W[A,E,I,K,L,P,Q,T,V]RE.[A,C,D,G,S,Y]A[C,D,F,G,H,I,L,N,R,S,V,Y]LRR[A,C,F,G,I,L,P,R,S,T,V,]AD[D,E,H,I,K,L,M,N,Q,V][A,D,F,H,I,L,N,P,S,T,V,Y]NAQ[A,F,I,L,P,S,T,V]|W[A,E,I,K,L,P,Q,T,V]REI.A[C,D,F,G,H,I,L,N,R,S,V,Y]LRR[A,C,F,G,I,L,P,R,S,T,V,]AD[D,E,H,I,K,L,M,N,Q,V][A,D,F,H,I,L,N,P,S,T,V,Y]NAQ[A,F,I,L,P,S,T,V]|W[A,E,I,K,L,P,Q,T,V]REI[A,C,D,G,S,Y].[C,D,F,G,H,I,L,N,R,S,V,Y]LRR[A,C,F,G,I,L,P,R,S,T,V,]AD[D,E,H,I,K,L,M,N,Q,V][A,D,F,H,I,L,N,P,S,T,V,Y]NAQ[A,F,I,L,P,S,T,V]|W[A,E,I,K,L,P,Q,T,V]REI[A,C,D,G,S,Y]A.LRR[A,C,F,G,I,L,P,R,S,T,V,]AD[D,E,H,I,K,L,M,N,Q,V][A,D,F,H,I,L,N,P,S,T,V,Y]NAQ[A,F,I,L,P,S,T,V]|W[A,E,I,K,L,P,Q,T,V]REI[A,C,D,G,S,Y]A[C,D,F,G,H,I,L,N,R,S,V,Y].RR[A,C,F,G,I,L,P,R,S,T,V,]AD[D,E,H,I,K,L,M,N,Q,V][A,D,F,H,I,L,N,P,S,T,V,Y]NAQ[A,F,I,L,P,S,T,V]|W[A,E,I,K,L,P,Q,T,V]REI[A,C,D,G,S,Y]A[C,D,F,G,H,I,L,N,R,S,V,Y]L.R[A,C,F,G,I,L,P,R,S,T,V,]AD[D,E,H,I,K,L,M,N,Q,V][A,D,F,H,I,L,N,P,S,T,V,Y]NAQ[A,F,I,L,P,S,T,V]|W[A,E,I,K,L,P,Q,T,V]REI[A,C,D,G,S,Y]A[C,D,F,G,H,I,L,N,R,S,V,Y]LR.[A,C,F,G,I,L,P,R,S,T,V,]AD[D,E,H,I,K,L,M,N,Q,V][A,D,F,H,I,L,N,P,S,T,V,Y]NAQ[A,F,I,L,P,S,T,V]|W[A,E,I,K,L,P,Q,T,V]REI[A,C,D,G,S,Y]A[C,D,F,G,H,I,L,N,R,S,V,Y]LRR.AD[D,E,H,I,K,L,M,N,Q,V][A,D,F,H,I,L,N,P,S,T,V,Y]NAQ[A,F,I,L,P,S,T,V]|W[A,E,I,K,L,P,Q,T,V]REI[A,C,D,G,S,Y]A[C,D,F,G,H,I,L,N,R,S,V,Y]LRR[A,C,F,G,I,L,P,R,S,T,V,].D[D,E,H,I,K,L,M,N,Q,V][A,D,F,H,I,L,N,P,S,T,V,Y]NAQ[A,F,I,L,P,S,T,V]|W[A,E,I,K,L,P,Q,T,V]REI[A,C,D,G,S,Y]A[C,D,F,G,H,I,L,N,R,S,V,Y]LRR[A,C,F,G,I,L,P,R,S,T,V,]A.[D,E,H,I,K,L,M,N,Q,V][A,D,F,H,I,L,N,P,S,T,V,Y]NAQ[A,F,I,L,P,S,T,V]|W[A,E,I,K,L,P,Q,T,V]REI[A,C,D,G,S,Y]A[C,D,F,G,H,I,L,N,R,S,V,Y]LRR[A,C,F,G,I,L,P,R,S,T,V,]AD.NAQ[A,F,I,L,P,S,T,V]|W[A,E,I,K,L,P,Q,T,V]REI[A,C,D,G,S,Y]A[C,D,F,G,H,I,L,N,R,S,V,Y]LRR[A,C,F,G,I,L,P,R,S,T,V,]AD[D,E,H,I,K,L,M,N,Q,V][A,D,F,H,I,L,N,P,S,T,V,Y].AQ[A,F,I,L,P,S,T,V]|W[A,E,I,K,L,P,Q,T,V]REI[A,C,D,G,S,Y]A[C,D,F,G,H,I,L,N,R,S,V,Y]LRR[A,C,F,G,I,L,P,R,S,T,V,]AD[D,E,H,I,K,L,M,N,Q,V][A,D,F,H,I,L,N,P,S,T,V,Y]N.Q[A,F,I,L,P,S,T,V]|W[A,E,I,K,L,P,Q,T,V]REI[A,C,D,G,S,Y]A[C,D,F,G,H,I,L,N,R,S,V,Y]LRR[A,C,F,G,I,L,P,R,S,T,V,]AD[D,E,H,I,K,L,M,N,Q,V][A,D,F,H,I,L,N,P,S,T,V,Y]NA.[A,F,I,L,P,S,T,V]|W[A,E,I,K,L,P,Q,T,V]REI[A,C,D,G,S,Y]A[C,D,F,G,H,I,L,N,R,S,V,Y]LRR[A,C,F,G,I,L,P,R,S,T,V,]AD[D,E,H,I,K,L,M,N,Q,V][A,D,F,H,I,L,N,P,S,T,V,Y]NAQ."),"lib"]='f'

#Assign unique sequences to Bcl-xl, Mcl-1, or Bfl-1 library from initial pool. Allows for up to one amino acid mutation from theoretical library. 
naivelib=pool
naivelib['rank'] = naivelib['count'].rank(ascending=0)
naivelib['lib'] = 'o'

naivelib.loc[naivelib['seq'].str.contains(".[A,E,I,K,L,P,Q,T,V]REI[A,C,D,G,S,Y]A[C,D,F,G,H,I,L,N,R,S,V,Y]LRR[A,C,F,G,I,L,P,R,S,T,V,]AD[D,E,H,I,K,L,M,N,Q,V][A,D,F,H,I,L,N,P,S,T,V,Y]NAQ[A,F,I,L,P,S,T,V]|W.REI[A,C,D,G,S,Y]A[C,D,F,G,H,I,L,N,R,S,V,Y]LRR[A,C,F,G,I,L,P,R,S,T,V,]AD[D,E,H,I,K,L,M,N,Q,V][A,D,F,H,I,L,N,P,S,T,V,Y]NAQ[A,F,I,L,P,S,T,V]|W[A,E,I,K,L,P,Q,T,V].EI[A,C,D,G,S,Y]A[C,D,F,G,H,I,L,N,R,S,V,Y]LRR[A,C,F,G,I,L,P,R,S,T,V,]AD[D,E,H,I,K,L,M,N,Q,V][A,D,F,H,I,L,N,P,S,T,V,Y]NAQ[A,F,I,L,P,S,T,V]|W[A,E,I,K,L,P,Q,T,V]R.I[A,C,D,G,S,Y]A[C,D,F,G,H,I,L,N,R,S,V,Y]LRR[A,C,F,G,I,L,P,R,S,T,V,]AD[D,E,H,I,K,L,M,N,Q,V][A,D,F,H,I,L,N,P,S,T,V,Y]NAQ[A,F,I,L,P,S,T,V]|W[A,E,I,K,L,P,Q,T,V]RE.[A,C,D,G,S,Y]A[C,D,F,G,H,I,L,N,R,S,V,Y]LRR[A,C,F,G,I,L,P,R,S,T,V,]AD[D,E,H,I,K,L,M,N,Q,V][A,D,F,H,I,L,N,P,S,T,V,Y]NAQ[A,F,I,L,P,S,T,V]|W[A,E,I,K,L,P,Q,T,V]REI.A[C,D,F,G,H,I,L,N,R,S,V,Y]LRR[A,C,F,G,I,L,P,R,S,T,V,]AD[D,E,H,I,K,L,M,N,Q,V][A,D,F,H,I,L,N,P,S,T,V,Y]NAQ[A,F,I,L,P,S,T,V]|W[A,E,I,K,L,P,Q,T,V]REI[A,C,D,G,S,Y].[C,D,F,G,H,I,L,N,R,S,V,Y]LRR[A,C,F,G,I,L,P,R,S,T,V,]AD[D,E,H,I,K,L,M,N,Q,V][A,D,F,H,I,L,N,P,S,T,V,Y]NAQ[A,F,I,L,P,S,T,V]|W[A,E,I,K,L,P,Q,T,V]REI[A,C,D,G,S,Y]A.LRR[A,C,F,G,I,L,P,R,S,T,V,]AD[D,E,H,I,K,L,M,N,Q,V][A,D,F,H,I,L,N,P,S,T,V,Y]NAQ[A,F,I,L,P,S,T,V]|W[A,E,I,K,L,P,Q,T,V]REI[A,C,D,G,S,Y]A[C,D,F,G,H,I,L,N,R,S,V,Y].RR[A,C,F,G,I,L,P,R,S,T,V,]AD[D,E,H,I,K,L,M,N,Q,V][A,D,F,H,I,L,N,P,S,T,V,Y]NAQ[A,F,I,L,P,S,T,V]|W[A,E,I,K,L,P,Q,T,V]REI[A,C,D,G,S,Y]A[C,D,F,G,H,I,L,N,R,S,V,Y]L.R[A,C,F,G,I,L,P,R,S,T,V,]AD[D,E,H,I,K,L,M,N,Q,V][A,D,F,H,I,L,N,P,S,T,V,Y]NAQ[A,F,I,L,P,S,T,V]|W[A,E,I,K,L,P,Q,T,V]REI[A,C,D,G,S,Y]A[C,D,F,G,H,I,L,N,R,S,V,Y]LR.[A,C,F,G,I,L,P,R,S,T,V,]AD[D,E,H,I,K,L,M,N,Q,V][A,D,F,H,I,L,N,P,S,T,V,Y]NAQ[A,F,I,L,P,S,T,V]|W[A,E,I,K,L,P,Q,T,V]REI[A,C,D,G,S,Y]A[C,D,F,G,H,I,L,N,R,S,V,Y]LRR.AD[D,E,H,I,K,L,M,N,Q,V][A,D,F,H,I,L,N,P,S,T,V,Y]NAQ[A,F,I,L,P,S,T,V]|W[A,E,I,K,L,P,Q,T,V]REI[A,C,D,G,S,Y]A[C,D,F,G,H,I,L,N,R,S,V,Y]LRR[A,C,F,G,I,L,P,R,S,T,V,].D[D,E,H,I,K,L,M,N,Q,V][A,D,F,H,I,L,N,P,S,T,V,Y]NAQ[A,F,I,L,P,S,T,V]|W[A,E,I,K,L,P,Q,T,V]REI[A,C,D,G,S,Y]A[C,D,F,G,H,I,L,N,R,S,V,Y]LRR[A,C,F,G,I,L,P,R,S,T,V,]A.[D,E,H,I,K,L,M,N,Q,V][A,D,F,H,I,L,N,P,S,T,V,Y]NAQ[A,F,I,L,P,S,T,V]|W[A,E,I,K,L,P,Q,T,V]REI[A,C,D,G,S,Y]A[C,D,F,G,H,I,L,N,R,S,V,Y]LRR[A,C,F,G,I,L,P,R,S,T,V,]AD.NAQ[A,F,I,L,P,S,T,V]|W[A,E,I,K,L,P,Q,T,V]REI[A,C,D,G,S,Y]A[C,D,F,G,H,I,L,N,R,S,V,Y]LRR[A,C,F,G,I,L,P,R,S,T,V,]AD[D,E,H,I,K,L,M,N,Q,V][A,D,F,H,I,L,N,P,S,T,V,Y].AQ[A,F,I,L,P,S,T,V]|W[A,E,I,K,L,P,Q,T,V]REI[A,C,D,G,S,Y]A[C,D,F,G,H,I,L,N,R,S,V,Y]LRR[A,C,F,G,I,L,P,R,S,T,V,]AD[D,E,H,I,K,L,M,N,Q,V][A,D,F,H,I,L,N,P,S,T,V,Y]N.Q[A,F,I,L,P,S,T,V]|W[A,E,I,K,L,P,Q,T,V]REI[A,C,D,G,S,Y]A[C,D,F,G,H,I,L,N,R,S,V,Y]LRR[A,C,F,G,I,L,P,R,S,T,V,]AD[D,E,H,I,K,L,M,N,Q,V][A,D,F,H,I,L,N,P,S,T,V,Y]NA.[A,F,I,L,P,S,T,V]|W[A,E,I,K,L,P,Q,T,V]REI[A,C,D,G,S,Y]A[C,D,F,G,H,I,L,N,R,S,V,Y]LRR[A,C,F,G,I,L,P,R,S,T,V,]AD[D,E,H,I,K,L,M,N,Q,V][A,D,F,H,I,L,N,P,S,T,V,Y]NAQ."),"lib"]='f'
naivelib.loc[naivelib['seq'].str.contains(".[DEHIKLMNQV]RE[DFHILNVY]GAQL[AGIRTV]R[ACDFGHILNPRSTVY][AG]DDL[ADHILNPTV][AEKT]Q[ACDGHNPRSTY]|W.RE[DFHILNVY]GAQL[AGIRTV]R[ACDFGHILNPRSTVY][AG]DDL[ADHILNPTV][AEKT]Q[ACDGHNPRSTY]|W[DEHIKLMNQV].E[DFHILNVY]GAQL[AGIRTV]R[ACDFGHILNPRSTVY][AG]DDL[ADHILNPTV][AEKT]Q[ACDGHNPRSTY]|W[DEHIKLMNQV]R.[DFHILNVY]GAQL[AGIRTV]R[ACDFGHILNPRSTVY][AG]DDL[ADHILNPTV][AEKT]Q[ACDGHNPRSTY]|W[DEHIKLMNQV]RE.GAQL[AGIRTV]R[ACDFGHILNPRSTVY][AG]DDL[ADHILNPTV][AEKT]Q[ACDGHNPRSTY]|W[DEHIKLMNQV]RE[DFHILNVY].AQL[AGIRTV]R[ACDFGHILNPRSTVY][AG]DDL[ADHILNPTV][AEKT]Q[ACDGHNPRSTY]|W[DEHIKLMNQV]RE[DFHILNVY]G.QL[AGIRTV]R[ACDFGHILNPRSTVY][AG]DDL[ADHILNPTV][AEKT]Q[ACDGHNPRSTY]|W[DEHIKLMNQV]RE[DFHILNVY]GA.L[AGIRTV]R[ACDFGHILNPRSTVY][AG]DDL[ADHILNPTV][AEKT]Q[ACDGHNPRSTY]|W[DEHIKLMNQV]RE[DFHILNVY]GAQ.[AGIRTV]R[ACDFGHILNPRSTVY][AG]DDL[ADHILNPTV][AEKT]Q[ACDGHNPRSTY]|W[DEHIKLMNQV]RE[DFHILNVY]GAQL.R[ACDFGHILNPRSTVY][AG]DDL[ADHILNPTV][AEKT]Q[ACDGHNPRSTY]|W[DEHIKLMNQV]RE[DFHILNVY]GAQL[AGIRTV].[ACDFGHILNPRSTVY][AG]DDL[ADHILNPTV][AEKT]Q[ACDGHNPRSTY]|W[DEHIKLMNQV]RE[DFHILNVY]GAQL[AGIRTV]R.[AG]DDL[ADHILNPTV][AEKT]Q[ACDGHNPRSTY]|W[DEHIKLMNQV]RE[DFHILNVY]GAQL[AGIRTV]R[ACDFGHILNPRSTVY].DDL[ADHILNPTV][AEKT]Q[ACDGHNPRSTY]|W[DEHIKLMNQV]RE[DFHILNVY]GAQL[AGIRTV]R[ACDFGHILNPRSTVY][AG].DL[ADHILNPTV][AEKT]Q[ACDGHNPRSTY]|W[DEHIKLMNQV]RE[DFHILNVY]GAQL[AGIRTV]R[ACDFGHILNPRSTVY][AG]D.L[ADHILNPTV][AEKT]Q[ACDGHNPRSTY]|W[DEHIKLMNQV]RE[DFHILNVY]GAQL[AGIRTV]R[ACDFGHILNPRSTVY][AG]DD.[ADHILNPTV][AEKT]Q[ACDGHNPRSTY]|W[DEHIKLMNQV]RE[DFHILNVY]GAQL[AGIRTV]R[ACDFGHILNPRSTVY][AG]DDL.[AEKT]Q[ACDGHNPRSTY]|W[DEHIKLMNQV]RE[DFHILNVY]GAQL[AGIRTV]R[ACDFGHILNPRSTVY][AG]DDL[ADHILNPTV].Q[ACDGHNPRSTY]|W[DEHIKLMNQV]RE[DFHILNVY]GAQL[AGIRTV]R[ACDFGHILNPRSTVY][AG]DDL[ADHILNPTV][AEKT].[ACDGHNPRSTY]|W[DEHIKLMNQV]RE[DFHILNVY]GAQL[AGIRTV]R[ACDFGHILNPRSTVY][AG]DDL[ADHILNPTV][AEKT]Q."),"lib"]='x'
naivelib.loc[naivelib['seq'].str.contains(".A[A,C,D,F,G,H,L,P,R,S,V,Y][D,E,H,Q][A,I,T,V][A,G,I,S,T,V]AQL[A,E,G,I,K,R,T,V]RMADD[A,D,F,H,I,L,N,P,S,T,V,Y][D,E,H,K,N,Q]AQY|[A,G,P,R,S,T,W].[A,C,D,F,G,H,L,P,R,S,V,Y][D,E,H,Q][A,I,T,V][A,G,I,S,T,V]AQL[A,E,G,I,K,R,T,V]RMADD[A,D,F,H,I,L,N,P,S,T,V,Y][D,E,H,K,N,Q]AQY|[A,G,P,R,S,T,W]A.[D,E,H,Q][A,I,T,V][A,G,I,S,T,V]AQL[A,E,G,I,K,R,T,V]RMADD[A,D,F,H,I,L,N,P,S,T,V,Y][D,E,H,K,N,Q]AQY|[A,G,P,R,S,T,W]A[A,C,D,F,G,H,L,P,R,S,V,Y].[A,I,T,V][A,G,I,S,T,V]AQL[A,E,G,I,K,R,T,V]RMADD[A,D,F,H,I,L,N,P,S,T,V,Y][D,E,H,K,N,Q]AQY|[A,G,P,R,S,T,W]A[A,C,D,F,G,H,L,P,R,S,V,Y][D,E,H,Q].[A,G,I,S,T,V]AQL[A,E,G,I,K,R,T,V]RMADD[A,D,F,H,I,L,N,P,S,T,V,Y][D,E,H,K,N,Q]AQY|[A,G,P,R,S,T,W]A[A,C,D,F,G,H,L,P,R,S,V,Y][D,E,H,Q][A,I,T,V].AQL[A,E,G,I,K,R,T,V]RMADD[A,D,F,H,I,L,N,P,S,T,V,Y][D,E,H,K,N,Q]AQY|[A,G,P,R,S,T,W]A[A,C,D,F,G,H,L,P,R,S,V,Y][D,E,H,Q][A,I,T,V][A,G,I,S,T,V].QL[A,E,G,I,K,R,T,V]RMADD[A,D,F,H,I,L,N,P,S,T,V,Y][D,E,H,K,N,Q]AQY|[A,G,P,R,S,T,W]A[A,C,D,F,G,H,L,P,R,S,V,Y][D,E,H,Q][A,I,T,V][A,G,I,S,T,V]A.L[A,E,G,I,K,R,T,V]RMADD[A,D,F,H,I,L,N,P,S,T,V,Y][D,E,H,K,N,Q]AQY|[A,G,P,R,S,T,W]A[A,C,D,F,G,H,L,P,R,S,V,Y][D,E,H,Q][A,I,T,V][A,G,I,S,T,V]AQ.[A,E,G,I,K,R,T,V]RMADD[A,D,F,H,I,L,N,P,S,T,V,Y][D,E,H,K,N,Q]AQY|[A,G,P,R,S,T,W]A[A,C,D,F,G,H,L,P,R,S,V,Y][D,E,H,Q][A,I,T,V][A,G,I,S,T,V]AQL.RMADD[A,D,F,H,I,L,N,P,S,T,V,Y][D,E,H,K,N,Q]AQY|[A,G,P,R,S,T,W]A[A,C,D,F,G,H,L,P,R,S,V,Y][D,E,H,Q][A,I,T,V][A,G,I,S,T,V]AQL[A,E,G,I,K,R,T,V].MADD[A,D,F,H,I,L,N,P,S,T,V,Y][D,E,H,K,N,Q]AQY|[A,G,P,R,S,T,W]A[A,C,D,F,G,H,L,P,R,S,V,Y][D,E,H,Q][A,I,T,V][A,G,I,S,T,V]AQL[A,E,G,I,K,R,T,V]R.ADD[A,D,F,H,I,L,N,P,S,T,V,Y][D,E,H,K,N,Q]AQY|[A,G,P,R,S,T,W]A[A,C,D,F,G,H,L,P,R,S,V,Y][D,E,H,Q][A,I,T,V][A,G,I,S,T,V]AQL[A,E,G,I,K,R,T,V]RM.DD[A,D,F,H,I,L,N,P,S,T,V,Y][D,E,H,K,N,Q]AQY|[A,G,P,R,S,T,W]A[A,C,D,F,G,H,L,P,R,S,V,Y][D,E,H,Q][A,I,T,V][A,G,I,S,T,V]AQL[A,E,G,I,K,R,T,V]RMA.D[A,D,F,H,I,L,N,P,S,T,V,Y][D,E,H,K,N,Q]AQY|[A,G,P,R,S,T,W]A[A,C,D,F,G,H,L,P,R,S,V,Y][D,E,H,Q][A,I,T,V][A,G,I,S,T,V]AQL[A,E,G,I,K,R,T,V]RMAD.[A,D,F,H,I,L,N,P,S,T,V,Y][D,E,H,K,N,Q]AQY|[A,G,P,R,S,T,W]A[A,C,D,F,G,H,L,P,R,S,V,Y][D,E,H,Q][A,I,T,V][A,G,I,S,T,V]AQL[A,E,G,I,K,R,T,V]RMADD.[D,E,H,K,N,Q]AQY|[A,G,P,R,S,T,W]A[A,C,D,F,G,H,L,P,R,S,V,Y][D,E,H,Q][A,I,T,V][A,G,I,S,T,V]AQL[A,E,G,I,K,R,T,V]RMADD[A,D,F,H,I,L,N,P,S,T,V,Y].AQY|[A,G,P,R,S,T,W]A[A,C,D,F,G,H,L,P,R,S,V,Y][D,E,H,Q][A,I,T,V][A,G,I,S,T,V]AQL[A,E,G,I,K,R,T,V]RMADD[A,D,F,H,I,L,N,P,S,T,V,Y][D,E,H,K,N,Q].QY|[A,G,P,R,S,T,W]A[A,C,D,F,G,H,L,P,R,S,V,Y][D,E,H,Q][A,I,T,V][A,G,I,S,T,V]AQL[A,E,G,I,K,R,T,V]RMADD[A,D,F,H,I,L,N,P,S,T,V,Y][D,E,H,K,N,Q]A.Y|[A,G,P,R,S,T,W]A[A,C,D,F,G,H,L,P,R,S,V,Y][D,E,H,Q][A,I,T,V][A,G,I,S,T,V]AQL[A,E,G,I,K,R,T,V]RMADD[A,D,F,H,I,L,N,P,S,T,V,Y][D,E,H,K,N,Q]AQ."),"lib"]='m'
```

In [19]:

```
#figure 3b
raw_data = {'Library': ['Bfl-1 Targeted', 'Mcl-1 Targeted', 'Bcl-xL Targeted'],
        'Final Pool': [len(LibcompRedo4noCnoB[LibcompRedo4noCnoB['lib']=='f']), len(LibcompRedo4noCnoB[LibcompRedo4noCnoB['lib']=='m']), len(LibcompRedo4noCnoB[LibcompRedo4noCnoB['lib']=='x'])],
        'Naive Pool': [len(naivelib[naivelib['lib']=='f']), len(naivelib[naivelib['lib']=='m']), len(naivelib[naivelib['lib']=='x'])]}
UniqueSequences = pd.DataFrame(raw_data, columns = ['Library', 'Naive Pool', 'Final Pool'])
plt.pie(UniqueSequences['Naive Pool'],
    shadow=False,
    startangle=90,
    colors=['#4878cf','#6acc65','#d65f5f'])
plt.axis('equal')
```

Out[19]:

```
(-1.002743039844151, 1.0078971041716627, -1.0049788084324305, 1.0)
```

In [20]:

```
#figure 3b
plt.pie(
    UniqueSequences['Final Pool'],
    shadow=False,
    startangle=90,
    colors=['#4878cf','#6acc65','#d65f5f']
    )
plt.axis('equal')
```

Out[20]:

```
(-1.0185668931451242, 1.0028528528490399, -1.0177011064865749, 1.0)
```

In [21]:

```
#Figure 3c
x = [-3,-2,-1,0, 1, 2, 3, 4, 5,6,7,8]# create line to indicate equal specifcity with PUMA
y = [-3,-2,-1,0, 1, 2, 3, 4, 5,6,7,8]
slope, intercept = np.polyfit(x, y, 1)
abline_values = [slope * i + intercept for i in x]

# STATIUM and SPOTpssm scores from seqences in the final pool from the Bfl-1 selective library.
CompRedotopFlib1mut=CompRedo4NoStopScoresAdj[CompRedo4NoStopScoresAdj['Sequence'].str.contains(".[A,E,I,K,L,P,Q,T,V]REI[A,C,D,G,S,Y]A[C,D,F,G,H,I,L,N,R,S,V,Y]LRR[A,C,F,G,I,L,P,R,S,T,V,]AD[D,E,H,I,K,L,M,N,Q,V][A,D,F,H,I,L,N,P,S,T,V,Y]NAQ[A,F,I,L,P,S,T,V]|W.REI[A,C,D,G,S,Y]A[C,D,F,G,H,I,L,N,R,S,V,Y]LRR[A,C,F,G,I,L,P,R,S,T,V,]AD[D,E,H,I,K,L,M,N,Q,V][A,D,F,H,I,L,N,P,S,T,V,Y]NAQ[A,F,I,L,P,S,T,V]|W[A,E,I,K,L,P,Q,T,V].EI[A,C,D,G,S,Y]A[C,D,F,G,H,I,L,N,R,S,V,Y]LRR[A,C,F,G,I,L,P,R,S,T,V,]AD[D,E,H,I,K,L,M,N,Q,V][A,D,F,H,I,L,N,P,S,T,V,Y]NAQ[A,F,I,L,P,S,T,V]|W[A,E,I,K,L,P,Q,T,V]R.I[A,C,D,G,S,Y]A[C,D,F,G,H,I,L,N,R,S,V,Y]LRR[A,C,F,G,I,L,P,R,S,T,V,]AD[D,E,H,I,K,L,M,N,Q,V][A,D,F,H,I,L,N,P,S,T,V,Y]NAQ[A,F,I,L,P,S,T,V]|W[A,E,I,K,L,P,Q,T,V]RE.[A,C,D,G,S,Y]A[C,D,F,G,H,I,L,N,R,S,V,Y]LRR[A,C,F,G,I,L,P,R,S,T,V,]AD[D,E,H,I,K,L,M,N,Q,V][A,D,F,H,I,L,N,P,S,T,V,Y]NAQ[A,F,I,L,P,S,T,V]|W[A,E,I,K,L,P,Q,T,V]REI.A[C,D,F,G,H,I,L,N,R,S,V,Y]LRR[A,C,F,G,I,L,P,R,S,T,V,]AD[D,E,H,I,K,L,M,N,Q,V][A,D,F,H,I,L,N,P,S,T,V,Y]NAQ[A,F,I,L,P,S,T,V]|W[A,E,I,K,L,P,Q,T,V]REI[A,C,D,G,S,Y].[C,D,F,G,H,I,L,N,R,S,V,Y]LRR[A,C,F,G,I,L,P,R,S,T,V,]AD[D,E,H,I,K,L,M,N,Q,V][A,D,F,H,I,L,N,P,S,T,V,Y]NAQ[A,F,I,L,P,S,T,V]|W[A,E,I,K,L,P,Q,T,V]REI[A,C,D,G,S,Y]A.LRR[A,C,F,G,I,L,P,R,S,T,V,]AD[D,E,H,I,K,L,M,N,Q,V][A,D,F,H,I,L,N,P,S,T,V,Y]NAQ[A,F,I,L,P,S,T,V]|W[A,E,I,K,L,P,Q,T,V]REI[A,C,D,G,S,Y]A[C,D,F,G,H,I,L,N,R,S,V,Y].RR[A,C,F,G,I,L,P,R,S,T,V,]AD[D,E,H,I,K,L,M,N,Q,V][A,D,F,H,I,L,N,P,S,T,V,Y]NAQ[A,F,I,L,P,S,T,V]|W[A,E,I,K,L,P,Q,T,V]REI[A,C,D,G,S,Y]A[C,D,F,G,H,I,L,N,R,S,V,Y]L.R[A,C,F,G,I,L,P,R,S,T,V,]AD[D,E,H,I,K,L,M,N,Q,V][A,D,F,H,I,L,N,P,S,T,V,Y]NAQ[A,F,I,L,P,S,T,V]|W[A,E,I,K,L,P,Q,T,V]REI[A,C,D,G,S,Y]A[C,D,F,G,H,I,L,N,R,S,V,Y]LR.[A,C,F,G,I,L,P,R,S,T,V,]AD[D,E,H,I,K,L,M,N,Q,V][A,D,F,H,I,L,N,P,S,T,V,Y]NAQ[A,F,I,L,P,S,T,V]|W[A,E,I,K,L,P,Q,T,V]REI[A,C,D,G,S,Y]A[C,D,F,G,H,I,L,N,R,S,V,Y]LRR.AD[D,E,H,I,K,L,M,N,Q,V][A,D,F,H,I,L,N,P,S,T,V,Y]NAQ[A,F,I,L,P,S,T,V]|W[A,E,I,K,L,P,Q,T,V]REI[A,C,D,G,S,Y]A[C,D,F,G,H,I,L,N,R,S,V,Y]LRR[A,C,F,G,I,L,P,R,S,T,V,].D[D,E,H,I,K,L,M,N,Q,V][A,D,F,H,I,L,N,P,S,T,V,Y]NAQ[A,F,I,L,P,S,T,V]|W[A,E,I,K,L,P,Q,T,V]REI[A,C,D,G,S,Y]A[C,D,F,G,H,I,L,N,R,S,V,Y]LRR[A,C,F,G,I,L,P,R,S,T,V,]A.[D,E,H,I,K,L,M,N,Q,V][A,D,F,H,I,L,N,P,S,T,V,Y]NAQ[A,F,I,L,P,S,T,V]|W[A,E,I,K,L,P,Q,T,V]REI[A,C,D,G,S,Y]A[C,D,F,G,H,I,L,N,R,S,V,Y]LRR[A,C,F,G,I,L,P,R,S,T,V,]AD.NAQ[A,F,I,L,P,S,T,V]|W[A,E,I,K,L,P,Q,T,V]REI[A,C,D,G,S,Y]A[C,D,F,G,H,I,L,N,R,S,V,Y]LRR[A,C,F,G,I,L,P,R,S,T,V,]AD[D,E,H,I,K,L,M,N,Q,V][A,D,F,H,I,L,N,P,S,T,V,Y].AQ[A,F,I,L,P,S,T,V]|W[A,E,I,K,L,P,Q,T,V]REI[A,C,D,G,S,Y]A[C,D,F,G,H,I,L,N,R,S,V,Y]LRR[A,C,F,G,I,L,P,R,S,T,V,]AD[D,E,H,I,K,L,M,N,Q,V][A,D,F,H,I,L,N,P,S,T,V,Y]N.Q[A,F,I,L,P,S,T,V]|W[A,E,I,K,L,P,Q,T,V]REI[A,C,D,G,S,Y]A[C,D,F,G,H,I,L,N,R,S,V,Y]LRR[A,C,F,G,I,L,P,R,S,T,V,]AD[D,E,H,I,K,L,M,N,Q,V][A,D,F,H,I,L,N,P,S,T,V,Y]NA.[A,F,I,L,P,S,T,V]|W[A,E,I,K,L,P,Q,T,V]REI[A,C,D,G,S,Y]A[C,D,F,G,H,I,L,N,R,S,V,Y]LRR[A,C,F,G,I,L,P,R,S,T,V,]AD[D,E,H,I,K,L,M,N,Q,V][A,D,F,H,I,L,N,P,S,T,V,Y]NAQ.")]                                                  

f, ax = plt.subplots(1, 1,figsize=(6, 4))
ax=sns.kdeplot(F100klibZScores['PSSM Bcl-xl'], F100klibZScores['PSSM Bfl1'],cmap="Blues_d")
ax.scatter(CompRedotopFlib1mut['PSSM Bcl-xl'], CompRedotopFlib1mut['PSSM Bfl1'],color='red',s=5)

ax.plot(x, abline_values, 'k--')
ax.set_xlim([-4.5,0.5])
ax.set_ylim([-2.5,1])
ax.set_xticks([-4,-3,-2,-1,0])
ax.set_yticks([-2,-1,0,1])
ax.set_xlabel('Bcl-x$_L$ PSSM score', size =24)
ax.set_ylabel('Bfl-1 PSSM score', size =24)
f.subplots_adjust(wspace=0)
ax.tick_params(axis='x', labelsize=18)
ax.tick_params(axis='y', labelsize=18)
sns.despine()
```

In [22]:

```
#Figure 3d
x = [-3,-2,-1,0, 1, 2, 3, 4, 5,6,7,8]# create line to indicate equal specifcity with PUMA
y = [-3,-2,-1,0, 1, 2, 3, 4, 5,6,7,8]
slope, intercept = np.polyfit(x, y, 1)
abline_values = [slope * i + intercept for i in x]

f, ax = plt.subplots(1, 1,figsize=(6, 4))
ax=sns.kdeplot(F100klibZScores['PSSM Bcl-xl'], F100klibZScores['PSSM Bfl1'],cmap="Blues_d")
ax.scatter(CompRedotopFlib1mut['PSSM Bcl-xl'], CompRedotopFlib1mut['PSSM Bfl1'],color='red',s=5)

ax.plot(x, abline_values, 'k--')
ax.set_xlim([-4.5,0.5])
ax.set_ylim([-2.5,1])
ax.set_xticks([-4,-3,-2,-1,0])
ax.set_yticks([-2,-1,0,1])
ax.set_xlabel('Bcl-x$_L$ PSSM score', size =24)
ax.set_ylabel('Bfl-1 PSSM score', size =24)
f.subplots_adjust(wspace=0)
ax.tick_params(axis='x', labelsize=18)
ax.tick_params(axis='y', labelsize=18)
sns.despine()
```

In [23]:

```
#Figure 3e
x = [-3,-2,-1,0, 1, 2, 3, 4, 5,6,7,8]+StandardsZScores.iloc[1]['Statium Raw Z Bcl-xl']-StandardsZScores.iloc[1]['Statium Raw Z Bfl1']
y = [-3,-2,-1,0, 1, 2, 3, 4, 5,6,7,8]# create line to indicate equal specifcity with PUMA
slope, intercept = np.polyfit(x, y, 1)
abline_values = [slope * i + intercept for i in x]

f, ax = plt.subplots(1, 1,figsize=(6, 4))
ax=sns.kdeplot(F100klibZScores['Statium Raw Z Bcl-xl'], F100klibZScores['Statium Raw Z Bfl1'],cmap="Blues_d")
ax.scatter(CompRedotopFlib1mut['Statium Raw Z Bcl-xl'], CompRedotopFlib1mut['Statium Raw Z Bfl1'],color='red',s=5)

ax.plot(x, abline_values, 'k--')
ax.set_xlim([-.5,4.5])
ax.set_ylim([0,4])
ax.set_xticks([0,1,2,3,4])
ax.set_yticks([1,2,3,4])
ax.set_xlabel('Bcl-x$_L$ STATIUM z-score', size =24)
ax.set_ylabel('Bfl-1 STATIUM z-score', size =24)

f.subplots_adjust(wspace=0)
ax.tick_params(axis='x', labelsize=18)
ax.tick_params(axis='y', labelsize=18)
sns.despine()
```

In [24]:

```
#Figure 3f
x = [-3,-2,-1,0, 1, 2, 3, 4, 5,6,7,8]+StandardsZScores.iloc[1]['Statium Raw Z Mcl1']-StandardsZScores.iloc[1]['Statium Raw Z Bfl1']
y = [-3,-2,-1,0, 1, 2, 3, 4, 5,6,7,8]# create line to indicate equal specifcity with PUMA
slope, intercept = np.polyfit(x, y, 1)
abline_values = [slope * i + intercept for i in x]

f, ax2 = plt.subplots(1, 1,figsize=(6, 4))
ax2=sns.kdeplot(F100klibZScores['Statium Raw Z Mcl1'], F100klibZScores['Statium Raw Z Bfl1'],cmap="Blues_d")
ax2.scatter(CompRedotopFlib1mut['Statium Raw Z Mcl1'], CompRedotopFlib1mut['Statium Raw Z Bfl1'],color='red',s=5)

ax2.plot(x, abline_values, 'k--')
ax2.set_xlim([-.5,4.5])
ax2.set_ylim([0,4])
ax2.set_xticks([0,1,2,3,4])
ax2.set_yticks([1,2,3,4])
ax2.set_xlabel('Mcl-1 STATIUM z-score', size =24)
ax2.set_ylabel('', size =24)
ax2.tick_params(axis='x', labelsize=18)
ax2.tick_params(axis='y', labelsize=0)
sns.despine()
```

In [25]:

```
from weblogolib import *
from IPython.display import Image
```

In [26]:

```
def makeLogo(sequences):
    myFile = open("/tmp/seqlogo",'w')
    for each in sequences:
        myFile.write(each)
        myFile.write("\n")
    myFile.close()
    myFile=open("/tmp/seqlogo",'r')
    seqs = read_seq_data(myFile)
    data = LogoData.from_seqs(seqs)
    options = LogoOptions()
    options.fineprint=""
    options.resolution=200
    options.fontsize=8
    options.unit_name="probability"
    myFormat = LogoFormat(data, options)
    return png_formatter(data,myFormat)
```

In [27]:

```
#Figure 4a
FcompRedo4noCnoB=compRedo4noCnoB[compRedo4noCnoB['seq'].str.contains(".[A,E,I,K,L,P,Q,T,V]REI[A,C,D,G,S,Y]A[C,D,F,G,H,I,L,N,R,S,V,Y]LRR[A,C,F,G,I,L,P,R,S,T,V,]AD[D,E,H,I,K,L,M,N,Q,V][A,D,F,H,I,L,N,P,S,T,V,Y]NAQ[A,F,I,L,P,S,T,V]|W.REI[A,C,D,G,S,Y]A[C,D,F,G,H,I,L,N,R,S,V,Y]LRR[A,C,F,G,I,L,P,R,S,T,V,]AD[D,E,H,I,K,L,M,N,Q,V][A,D,F,H,I,L,N,P,S,T,V,Y]NAQ[A,F,I,L,P,S,T,V]|W[A,E,I,K,L,P,Q,T,V].EI[A,C,D,G,S,Y]A[C,D,F,G,H,I,L,N,R,S,V,Y]LRR[A,C,F,G,I,L,P,R,S,T,V,]AD[D,E,H,I,K,L,M,N,Q,V][A,D,F,H,I,L,N,P,S,T,V,Y]NAQ[A,F,I,L,P,S,T,V]|W[A,E,I,K,L,P,Q,T,V]R.I[A,C,D,G,S,Y]A[C,D,F,G,H,I,L,N,R,S,V,Y]LRR[A,C,F,G,I,L,P,R,S,T,V,]AD[D,E,H,I,K,L,M,N,Q,V][A,D,F,H,I,L,N,P,S,T,V,Y]NAQ[A,F,I,L,P,S,T,V]|W[A,E,I,K,L,P,Q,T,V]RE.[A,C,D,G,S,Y]A[C,D,F,G,H,I,L,N,R,S,V,Y]LRR[A,C,F,G,I,L,P,R,S,T,V,]AD[D,E,H,I,K,L,M,N,Q,V][A,D,F,H,I,L,N,P,S,T,V,Y]NAQ[A,F,I,L,P,S,T,V]|W[A,E,I,K,L,P,Q,T,V]REI.A[C,D,F,G,H,I,L,N,R,S,V,Y]LRR[A,C,F,G,I,L,P,R,S,T,V,]AD[D,E,H,I,K,L,M,N,Q,V][A,D,F,H,I,L,N,P,S,T,V,Y]NAQ[A,F,I,L,P,S,T,V]|W[A,E,I,K,L,P,Q,T,V]REI[A,C,D,G,S,Y].[C,D,F,G,H,I,L,N,R,S,V,Y]LRR[A,C,F,G,I,L,P,R,S,T,V,]AD[D,E,H,I,K,L,M,N,Q,V][A,D,F,H,I,L,N,P,S,T,V,Y]NAQ[A,F,I,L,P,S,T,V]|W[A,E,I,K,L,P,Q,T,V]REI[A,C,D,G,S,Y]A.LRR[A,C,F,G,I,L,P,R,S,T,V,]AD[D,E,H,I,K,L,M,N,Q,V][A,D,F,H,I,L,N,P,S,T,V,Y]NAQ[A,F,I,L,P,S,T,V]|W[A,E,I,K,L,P,Q,T,V]REI[A,C,D,G,S,Y]A[C,D,F,G,H,I,L,N,R,S,V,Y].RR[A,C,F,G,I,L,P,R,S,T,V,]AD[D,E,H,I,K,L,M,N,Q,V][A,D,F,H,I,L,N,P,S,T,V,Y]NAQ[A,F,I,L,P,S,T,V]|W[A,E,I,K,L,P,Q,T,V]REI[A,C,D,G,S,Y]A[C,D,F,G,H,I,L,N,R,S,V,Y]L.R[A,C,F,G,I,L,P,R,S,T,V,]AD[D,E,H,I,K,L,M,N,Q,V][A,D,F,H,I,L,N,P,S,T,V,Y]NAQ[A,F,I,L,P,S,T,V]|W[A,E,I,K,L,P,Q,T,V]REI[A,C,D,G,S,Y]A[C,D,F,G,H,I,L,N,R,S,V,Y]LR.[A,C,F,G,I,L,P,R,S,T,V,]AD[D,E,H,I,K,L,M,N,Q,V][A,D,F,H,I,L,N,P,S,T,V,Y]NAQ[A,F,I,L,P,S,T,V]|W[A,E,I,K,L,P,Q,T,V]REI[A,C,D,G,S,Y]A[C,D,F,G,H,I,L,N,R,S,V,Y]LRR.AD[D,E,H,I,K,L,M,N,Q,V][A,D,F,H,I,L,N,P,S,T,V,Y]NAQ[A,F,I,L,P,S,T,V]|W[A,E,I,K,L,P,Q,T,V]REI[A,C,D,G,S,Y]A[C,D,F,G,H,I,L,N,R,S,V,Y]LRR[A,C,F,G,I,L,P,R,S,T,V,].D[D,E,H,I,K,L,M,N,Q,V][A,D,F,H,I,L,N,P,S,T,V,Y]NAQ[A,F,I,L,P,S,T,V]|W[A,E,I,K,L,P,Q,T,V]REI[A,C,D,G,S,Y]A[C,D,F,G,H,I,L,N,R,S,V,Y]LRR[A,C,F,G,I,L,P,R,S,T,V,]A.[D,E,H,I,K,L,M,N,Q,V][A,D,F,H,I,L,N,P,S,T,V,Y]NAQ[A,F,I,L,P,S,T,V]|W[A,E,I,K,L,P,Q,T,V]REI[A,C,D,G,S,Y]A[C,D,F,G,H,I,L,N,R,S,V,Y]LRR[A,C,F,G,I,L,P,R,S,T,V,]AD.NAQ[A,F,I,L,P,S,T,V]|W[A,E,I,K,L,P,Q,T,V]REI[A,C,D,G,S,Y]A[C,D,F,G,H,I,L,N,R,S,V,Y]LRR[A,C,F,G,I,L,P,R,S,T,V,]AD[D,E,H,I,K,L,M,N,Q,V][A,D,F,H,I,L,N,P,S,T,V,Y].AQ[A,F,I,L,P,S,T,V]|W[A,E,I,K,L,P,Q,T,V]REI[A,C,D,G,S,Y]A[C,D,F,G,H,I,L,N,R,S,V,Y]LRR[A,C,F,G,I,L,P,R,S,T,V,]AD[D,E,H,I,K,L,M,N,Q,V][A,D,F,H,I,L,N,P,S,T,V,Y]N.Q[A,F,I,L,P,S,T,V]|W[A,E,I,K,L,P,Q,T,V]REI[A,C,D,G,S,Y]A[C,D,F,G,H,I,L,N,R,S,V,Y]LRR[A,C,F,G,I,L,P,R,S,T,V,]AD[D,E,H,I,K,L,M,N,Q,V][A,D,F,H,I,L,N,P,S,T,V,Y]NA.[A,F,I,L,P,S,T,V]|W[A,E,I,K,L,P,Q,T,V]REI[A,C,D,G,S,Y]A[C,D,F,G,H,I,L,N,R,S,V,Y]LRR[A,C,F,G,I,L,P,R,S,T,V,]AD[D,E,H,I,K,L,M,N,Q,V][A,D,F,H,I,L,N,P,S,T,V,Y]NAQ.")]
print len(FcompRedo4noCnoB)
Image(makeLogo(list(FcompRedo4noCnoB['seq'])))
```

```
612
```

Out[27]:

In [28]:

```
#load data sets that have been scored with STATIUM on the Bfl-1:FS2 structure. 
CompRedo4ZScores = pd.read_csv("/Users/jmjenson/Desktop/FSpaper/LibDesign/compRedo4Zcomplete.txt")
#calculate differences in STATIUM scores between original model and Bfl-1:FS2 complex
CompRedo4ZScores['diffFS2orig']=CompRedo4ZScores['Statium Raw Z Bfl1 FS2']-CompRedo4ZScores['Statium Raw Z Bfl1']
CompRedotopFlib1mut=CompRedo4ZScores[CompRedo4ZScores['Sequence'].str.contains(".[A,E,I,K,L,P,Q,T,V]REI[A,C,D,G,S,Y]A[C,D,F,G,H,I,L,N,R,S,V,Y]LRR[A,C,F,G,I,L,P,R,S,T,V,]AD[D,E,H,I,K,L,M,N,Q,V][A,D,F,H,I,L,N,P,S,T,V,Y]NAQ[A,F,I,L,P,S,T,V]|W.REI[A,C,D,G,S,Y]A[C,D,F,G,H,I,L,N,R,S,V,Y]LRR[A,C,F,G,I,L,P,R,S,T,V,]AD[D,E,H,I,K,L,M,N,Q,V][A,D,F,H,I,L,N,P,S,T,V,Y]NAQ[A,F,I,L,P,S,T,V]|W[A,E,I,K,L,P,Q,T,V].EI[A,C,D,G,S,Y]A[C,D,F,G,H,I,L,N,R,S,V,Y]LRR[A,C,F,G,I,L,P,R,S,T,V,]AD[D,E,H,I,K,L,M,N,Q,V][A,D,F,H,I,L,N,P,S,T,V,Y]NAQ[A,F,I,L,P,S,T,V]|W[A,E,I,K,L,P,Q,T,V]R.I[A,C,D,G,S,Y]A[C,D,F,G,H,I,L,N,R,S,V,Y]LRR[A,C,F,G,I,L,P,R,S,T,V,]AD[D,E,H,I,K,L,M,N,Q,V][A,D,F,H,I,L,N,P,S,T,V,Y]NAQ[A,F,I,L,P,S,T,V]|W[A,E,I,K,L,P,Q,T,V]RE.[A,C,D,G,S,Y]A[C,D,F,G,H,I,L,N,R,S,V,Y]LRR[A,C,F,G,I,L,P,R,S,T,V,]AD[D,E,H,I,K,L,M,N,Q,V][A,D,F,H,I,L,N,P,S,T,V,Y]NAQ[A,F,I,L,P,S,T,V]|W[A,E,I,K,L,P,Q,T,V]REI.A[C,D,F,G,H,I,L,N,R,S,V,Y]LRR[A,C,F,G,I,L,P,R,S,T,V,]AD[D,E,H,I,K,L,M,N,Q,V][A,D,F,H,I,L,N,P,S,T,V,Y]NAQ[A,F,I,L,P,S,T,V]|W[A,E,I,K,L,P,Q,T,V]REI[A,C,D,G,S,Y].[C,D,F,G,H,I,L,N,R,S,V,Y]LRR[A,C,F,G,I,L,P,R,S,T,V,]AD[D,E,H,I,K,L,M,N,Q,V][A,D,F,H,I,L,N,P,S,T,V,Y]NAQ[A,F,I,L,P,S,T,V]|W[A,E,I,K,L,P,Q,T,V]REI[A,C,D,G,S,Y]A.LRR[A,C,F,G,I,L,P,R,S,T,V,]AD[D,E,H,I,K,L,M,N,Q,V][A,D,F,H,I,L,N,P,S,T,V,Y]NAQ[A,F,I,L,P,S,T,V]|W[A,E,I,K,L,P,Q,T,V]REI[A,C,D,G,S,Y]A[C,D,F,G,H,I,L,N,R,S,V,Y].RR[A,C,F,G,I,L,P,R,S,T,V,]AD[D,E,H,I,K,L,M,N,Q,V][A,D,F,H,I,L,N,P,S,T,V,Y]NAQ[A,F,I,L,P,S,T,V]|W[A,E,I,K,L,P,Q,T,V]REI[A,C,D,G,S,Y]A[C,D,F,G,H,I,L,N,R,S,V,Y]L.R[A,C,F,G,I,L,P,R,S,T,V,]AD[D,E,H,I,K,L,M,N,Q,V][A,D,F,H,I,L,N,P,S,T,V,Y]NAQ[A,F,I,L,P,S,T,V]|W[A,E,I,K,L,P,Q,T,V]REI[A,C,D,G,S,Y]A[C,D,F,G,H,I,L,N,R,S,V,Y]LR.[A,C,F,G,I,L,P,R,S,T,V,]AD[D,E,H,I,K,L,M,N,Q,V][A,D,F,H,I,L,N,P,S,T,V,Y]NAQ[A,F,I,L,P,S,T,V]|W[A,E,I,K,L,P,Q,T,V]REI[A,C,D,G,S,Y]A[C,D,F,G,H,I,L,N,R,S,V,Y]LRR.AD[D,E,H,I,K,L,M,N,Q,V][A,D,F,H,I,L,N,P,S,T,V,Y]NAQ[A,F,I,L,P,S,T,V]|W[A,E,I,K,L,P,Q,T,V]REI[A,C,D,G,S,Y]A[C,D,F,G,H,I,L,N,R,S,V,Y]LRR[A,C,F,G,I,L,P,R,S,T,V,].D[D,E,H,I,K,L,M,N,Q,V][A,D,F,H,I,L,N,P,S,T,V,Y]NAQ[A,F,I,L,P,S,T,V]|W[A,E,I,K,L,P,Q,T,V]REI[A,C,D,G,S,Y]A[C,D,F,G,H,I,L,N,R,S,V,Y]LRR[A,C,F,G,I,L,P,R,S,T,V,]A.[D,E,H,I,K,L,M,N,Q,V][A,D,F,H,I,L,N,P,S,T,V,Y]NAQ[A,F,I,L,P,S,T,V]|W[A,E,I,K,L,P,Q,T,V]REI[A,C,D,G,S,Y]A[C,D,F,G,H,I,L,N,R,S,V,Y]LRR[A,C,F,G,I,L,P,R,S,T,V,]AD.NAQ[A,F,I,L,P,S,T,V]|W[A,E,I,K,L,P,Q,T,V]REI[A,C,D,G,S,Y]A[C,D,F,G,H,I,L,N,R,S,V,Y]LRR[A,C,F,G,I,L,P,R,S,T,V,]AD[D,E,H,I,K,L,M,N,Q,V][A,D,F,H,I,L,N,P,S,T,V,Y].AQ[A,F,I,L,P,S,T,V]|W[A,E,I,K,L,P,Q,T,V]REI[A,C,D,G,S,Y]A[C,D,F,G,H,I,L,N,R,S,V,Y]LRR[A,C,F,G,I,L,P,R,S,T,V,]AD[D,E,H,I,K,L,M,N,Q,V][A,D,F,H,I,L,N,P,S,T,V,Y]N.Q[A,F,I,L,P,S,T,V]|W[A,E,I,K,L,P,Q,T,V]REI[A,C,D,G,S,Y]A[C,D,F,G,H,I,L,N,R,S,V,Y]LRR[A,C,F,G,I,L,P,R,S,T,V,]AD[D,E,H,I,K,L,M,N,Q,V][A,D,F,H,I,L,N,P,S,T,V,Y]NA.[A,F,I,L,P,S,T,V]|W[A,E,I,K,L,P,Q,T,V]REI[A,C,D,G,S,Y]A[C,D,F,G,H,I,L,N,R,S,V,Y]LRR[A,C,F,G,I,L,P,R,S,T,V,]AD[D,E,H,I,K,L,M,N,Q,V][A,D,F,H,I,L,N,P,S,T,V,Y]NAQ.")]                                                  
F100klibZScores['diffFS2orig']=F100klibZScores['Statium Raw Z Bfl1 FS2'] -F100klibZScores['Statium Raw Z Bfl1']
```

In [29]:

```
#Figure 5g. 
f, ax1 = plt.subplots(1, 1,figsize=(6, 4))
weights = np.ones_like(CompRedotopFlib1mut['diffFS2orig'])/float(len(CompRedotopFlib1mut))
weights1 = np.ones_like(F100klibZScores['diffFS2orig'])/float(len(F100klibZScores))
ax1.hist(F100klibZScores['diffFS2orig'],bins=20,weights=weights1,range=[-1,2.5],label='Naive Lib',histtype='step',linewidth=2)
ax1.hist(CompRedotopFlib1mut['diffFS2orig'],bins=20,weights=weights,range=[-1,2.5],label='Final Pool',histtype='step',linewidth=2)
ax1.set_xticks([-1,0,1,2])
ax1.set_yticks([0,.05,.1,.15])
ax1.set_xlabel('(FS2-BID) STATIUM z-score', size=24)
ax1.set_ylabel('Frequency', size=24)
ax1.tick_params(axis='x', labelsize=18)
ax1.tick_params(axis='y', labelsize=18)
sns.despine()
```
